# Understanding the Shifts of Microbial Community and Metabolite Profile From Wheat to Mature *Daqu*

**DOI:** 10.3389/fmicb.2021.714726

**Published:** 2021-07-12

**Authors:** Yuandi Zhang, Yi Shen, Wei Cheng, Xi Wang, Yansong Xue, Xiaoxue Chen, Bei-Zhong Han

**Affiliations:** ^1^Beijing Laboratory for Food Quality and Safety, College of Food Science and Nutritional Engineering, China Agricultural University, Beijing, China; ^2^Sichuan Langjiu Co., Ltd., Luzhou, China

**Keywords:** *Daqu*, maturation, microbiota, metabolites, wheat

## Abstract

Wheat-originated microbes play an important role in shaping the quality of high-temperature *Daqu* which is commonly used as a starter for producing sauce-flavor *Baijiu*. However, the shifts of microbiota from raw material to fresh *Daqu* and then to mature *Daqu* remain unclear. Hence, in the present study, the inner and outer of fresh and mature *Daqu* were collected to explore the correlation between microbiota and metabolites as well as the source of the microbiota in *Daqu*. Results indicated that the activities of amylase and protease between the inner and outer of fresh *Daqu* varied significantly while both parts became similar after maturation. The predominant bacteria shifted from *Saccharopolyspora* (outer) and *Staphylococcus* (inner) to *Kroppenstedtia* (both outer and inner), while the predominant fungi shifted from *Thermoascus* (both outer and inner) to *Byssochlamys* (outer) and *Fusarium* (inner). A combining analysis of headspace solid-phase micro extraction-gas chromatography-mass spectrometry, headspace gas chromatography-ion mobility spectrometry, and nuclear magnetic resonance was employed to detect the metabolites. The network analysis was conducted to perform the relationships between microbes and metabolites. The results showed that the bacteria, especially *Saccharopolyspora*, *Bacillus*, and *Acinetobacter*, had a strong correlation with the productions of esters, amino acids and their derivatives, and sugars and their derivatives, while most fungi such as *Thermoascus*, were negatively correlated with the phenylalanine, trimethylamine n-oxide, and isovalerate. SourceTracker analysis indicated that wheat was the important source of the *Daqu* microbiota, especially, the microorganisms in the inner of *Daqu* might be the drivers of the microbial succession during maturation. This study provided a comprehensive exploration to understand the microbial sources and shifts in high-temperature *Daqu* during maturation.

## Introduction

*Baijiu* is a fermented alcoholic beverage and plays an important role in the history of Chinese food culture for 3000 years (Jin et al., 2017). The popularity of *Baijiu* to a great extent is due to its rich tastes and characterized flavors, both of which are produced in the complex production processes, including solid-state fermentation, distillation, storage, and blending (Xu et al., 2010). Particularly, fermentation plays an important role in the production of volatile flavors and critical secondary metabolites (Zheng et al., 2011). *Daqu*, made of wheat, barley, pea, and/or corn, is the most common starter and adopted in all the famous *Baijiu* in China (Wang et al., 2021). *Daqu* production needs a series of steps, including moistening, breaking, mixing shaping, spreading, incubating, and maturation (Zheng et al., 2011). Among them, maturation is the vital step that involves microbial enrichment, primary and secondary metabolic processes, and changes in diverse microbes and metabolites (Fan et al., 2019).

*Daqu* production still mainly relies on the experience of the operators to evaluate the quality of the raw materials and the maturation degree of the *Daqu*, which might not be objective all the time (Deng et al., 2020). The development of the new detection methods, like gas chromatography-mass spectrometry (GC-MS), high performance liquid chromatography-mass spectrometry (HPLC-MS), leads to the discrimination of *Daqu* maturation easier and more accurate to some extent (Pang et al., 2020). However, the microbiota is the original driver of the maturation. Maturation can promote enriched microbes to interact further, thereby leading to the mixed microbial communities and various metabolites to form balanced and stable *Daqu* (Gou et al., 2015). Culture-dependent and Illumina MiSeq sequencing methods were used to determine the microbial community during the maturation. A comprehensive understanding of the source and succession of the microbiota and the metabolites is very useful and urgent for the stable production of the high-quality *Daqu*.

Sauce-flavor *Baijiu*, one of the most popular *Baijiu* in China, is famous for its flavor resembling soy sauce, full-body, and long-lasting aroma (Zheng and Han, 2016). Its production relies on the high-temperature *Daqu* and the traditional production steps as shown in Supplementary Figure 1. Recently, microbiological and physicochemical methods were combined to explore the distribution of microbiota and various metabolites in *Daqu*. Jin et al. (2019) revealed the dominant microbes in *Daqu* and their distribution characteristics and detected the volatile flavors by headspace solid-phase micro extraction-gas chromatography-mass spectrometry (HS-SPME-GC-MS). Their results showed that the dominant bacteria of sauce-flavor *Daqu* were *Bacillales*, *Enterobacteriales*, and *Lactobacillales*, while the dominant fungi were *Trichoderma*, *Candida*, *Aspergillus*, *Thermomyces*, and *Trichosporon*. In Jin et al. (2019) study, the results showed that bacterial diversity was higher than fungal diversity in *Daqu*, and had the significant differences in microbial composition, distribution and physicochemical indices between the inner and outer parts of *Daqu*. These studies were all conducted on the mature *Daqu* and not associated with the changes during the maturation.

In the present study, Illumina MiSeq sequencing was used to investigate the microbiota succession from wheat, to fresh *Daqu* and mature *Daqu*. HS-SPME-GC-MS, headspace gas chromatography-ion mobility spectrometry (HS-GC-IMS), and nuclear magnetic resonance (^1^H NMR) were combined to comprehensively evaluate the metabolites in the different parts of *Daqu*, and different steps of *Daqu* production. Besides, SourceTracker analysis was employed to evaluate the contribution of wheat to *Daqu* in microbiota (Jin et al., 2019; Chen et al., 2021). It is the first comprehensive study on shifts in microbiota and metabolites for maturation of the sauce-flavor *Daqu* and will shed light on the improvement of *Daqu* production and the quality of the sauce-flavor *Baijiu*.

## Materials and Methods

### Sampling

The samples were collected from a sauce-flavor *Baijiu* manufacturer in Sichuan, China in 2020. Samples were collected in two different points: fresh *Daqu* and mature *Daqu*. Fresh *Daqu* (F-*Daqu*) refers to the newly made *Daqu* that needs 6 months of maturation. Mature *Daqu* (M-*Daqu*) refers to *Daqu* that is ready for *Baijiu* fermentation. To get reliable samples, *Daqu* bricks from each step were randomly selected from upper, middle, and lower locations in triplicate. Then each *Daqu* block was separated into two parts, the surface layer of 2.0∼3.0 cm thick was named the outer part of *Daqu* (O-*Daqu*), and the remaining central part was named the inner part of *Daqu* (I-*Daqu*). Raw material (wheat) samples (3.0 kg) were collected randomly from the storage. All samples were made in triplicate. *Daqu* samples were stored at 4°C for microbial counts and −20°C for metabolite analysis and Illumina MiSeq sequencing analysis.

### Microbiological Analysis

Total aerobic bacteria and thermophilic bacteria were enumerated on Plate Count Agar (PCA) (Aobox, Beijing, China), and were cultured at 37 and 55°C, respectively. Lactic acid bacteria (LAB) were enumerated on LAB culture medium (MRS; Aobox, Beijing, China) with natamycin 500 μg/ml. The plates were incubated at 30°C for 48–72 h (Pradhan and Tamang, 2019). Fungi were enumerated on two different media named Malt Extract Agar (MEA) (Aobox, Beijing, China) and Rose Bengal Chloramphenicol Agar (RBCA) (Aobox, Beijing, China), respectively, to which 100 mg/L chloramphenicol (Oxoid, SR0078E) was added. Colony Forming Units (CFU) were calculated and converted to the value of base 10 Logarithm and recorded as log_10_ (CFU/g) (Zheng et al., 2012).

### Analyses of Physicochemical Properties and Enzymology Properties

Moisture in *Daqu* samples was determined with a gravimetric method by drying 5.0 g samples at 105°C for 3 h (Pang et al., 2020). *Daqu* powder (5.0 g) was soaked in 50 mL of distilled water for 30 min, and water extracts were collected after filtration. The pH was measured using a pH meter positioned in the slurry (Zheng et al., 2012). The activity of glucoamylase and protease were determined according to the 3,5-dinitrosalicylic acid (DNS) method and Folin-phenol method, respectively (Fan G. S. et al., 2020). One unit of glucoamylase activity was defined as the amount of *Daqu* required for the liberation of 1 μmol glucose per minute in PBS buffer (50 mM, pH 6.5) at 40°C. One unit of protease activity was defined as the amount of *Daqu* required for the liberation of 1 mg tyrosine per minute in PBS buffer (50 mM, pH 7.0) at 40°C (Li et al., 2017).

### Analysis of Volatile Flavors by HS-SPME-GC-MS

The volatile flavors were analyzed by HS-SPME-GC-MS. The pre-treatment of HS-SPME-GC-MS was carried out as described below: 2.0 g sample was mixed with 8.0 mL Milli-Q water and subjected to ultrasonic treatment for 30 min (Liu et al., 2019; Pang et al., 2020). This was carried out in triplicates. Following this, the sample solutions were centrifuged at 6000 × *g* at 4°C for 15 min. Subsequently, 8 mL supernatant from the samples, and 2.0 g sodium chloride were placed into a 20 ml vial. This process was repeated with all three (triplicate) samples (Fan Y. et al., 2020). The volatile compounds were extracted with SPME fiber at 50°C for 50 min. The contents of volatile compounds were detected by GC-MS (Zhang et al., 2020).

### Analysis of Volatile Flavors by HS-GC-IMS

To investigate the spectrum changes of metabolites in *Daqu* samples, an untargeted analysis of volatile fingerprints was performed on a GC-IMS system (FlavourSpec^®^, Gesellschaft für Analytische Sensorsysteme mbH, Dortmund, Germany) equipped with an automatic headspace sampler unit (CTC-PAL, CTC Analytics AG, Zwingen, Switzerland). Extraction of volatile flavors from *Daqu* by SPME refers to the method of Yang et al. (2020) with minor modifications. In brief, 2.5 g of *Daqu* was weighed and placed into 20 mL headspace glass sampling vial. Subsequently, samples were incubated at 60°C for 15 min. After incubation, 200 μL of headspace was automatically injected into the injector. The volatile flavors from the *Daqu* were analyzed by GC-IMS referring to the method of Li et al. (2019) with minor modifications. The volatile flavors were separated and analyzed using a GC-IMS instrument, equipped with a quartz capillary column (FS-SE-54-CB-1, 15 m × 0.53 mm, 0.5 μm). The GC condition is as following: column temperature, 60°C; carrier gas: ultrapure nitrogen (purity ≥ 99.999%); carrier gas flow rate, 2 mL/min (0–2 min), 2–10 mL/min (2–10 min), 10–100 mL/min (10–20 min), 100–150 mL/min (20–30 min); detection time, 25 min (Yang et al., 2020). The following conditions were maintained in the automatic headspace sampler condition: incubation temperature, 60°C; incubation time, 15 min; injection method, headspace injection; injection volume, 200 μL; injection needle temperature, 85°C; heating method, shaking heating; shaking speed, 500 r/min; splitless injection (Márquez-Sillero et al., 2011). IMS detection condition is as follows: length of the drift tube, 98 mm; linear voltage in the tube, 500 V/cm; the temperature of drift tube, 45°C; drift gas, ultrapure nitrogen (purity ≥ 99.999%); the volume of drift gas, 150 mL/min; radioactive source, β-rays (tritium, 3H); ionization mode, positive ion (Yang et al., 2020).

### Analysis of Polar Metabolites by NMR

The aqueous extracts for NMR measurements were prepared as reported previously. A 600 μl aliquot of each sample was transferred into a 5-mm NMR tube. All ^1^H NMR spectra were measured at 300 K using an AVANCE NMR spectrometer (proton frequency 1/4 600.13 MHz, 14.1 T; Bruker, Billerica, Germany) with a cryogenic NMR probe (Wu et al., 2009). The ^1^H NMR experiments were performed using the following conditions: NOESYGPPRR1D pulse sequence; relaxation delay, 4.00 s; mixing time (for NOESY), 1.00 s; acquisition time, 2.28 s; number of steady states transients (dummy scans), 4; gradient pulse time, 1.00 ms; solvent suppression, pre-saturation with spoil gradient; spectral width, 7184 Hz; and time domain size, 32 k (Li et al., 2018). The compounds were identified and quantified with Chenomx software (version 7.6; Chenomx, Edmonton, Canada) with reference to the internal standard TSP. Each ^1^H NMR spectrum was equally divided into 242 fragments with width of 0.04 ppm. Spectra both with a range of 0.00∼10.00 ppm and exception of residual water resonance (4.50∼4.80 ppm), were divided into 0.04 ppm wide bins, followed by importing the achieved integral values into Microsoft^®^ Excel (Microsoft Corporation, Redmond, WA, United States) (Wu et al., 2009).

### DNA Extraction and Illumina MiSeq Sequencing Analysis

DNA extraction was carried out with E.Z.N.A.^®^ Soil DNA kits (Omega, Norcross, GA, United States) as per the manufacturer’s instructions. For bacteria, the V3-V4 domains of the 16 S rRNA genes were amplified using primers 338F and 806R. For fungi, the internal transcribed spacer (ITS) ITS1 regions were amplified with primers ZIT_F and ZITS_R (He et al., 2019). Reaction conditions consisted of an initial 95°C for 3 min followed by 35 cycles of 95°C for 30 s, 55°C for 30 s, and 72°C for 45 s, and a final extension of 72°C for 10 min. The purified amplicons were paired-end sequenced on an Illumina MiSeq PE300 system (Illumina, San Diego, CA, United States). Trimmomatic software (version 0.36) was used for mass filtering of the merged sequence (Bolger et al., 2014). Then Uchime software (version 8.1) was used to identify and remove chimeric sequences. Bioinformatics analysis of high-quality sequences was performed on a quantitative insight in microbial ecology (QIIME 1.8.0) platform. Clustering operation taxonomic units (OTUs) from clean tags with 97% similarity was done using the Uparse software (version 9.6). Then the taxonomic information was assigned to all bacterial OTUs via searching against Silva database (Release 132)^1^ and all fungal OTUs via searching against Unite (Release 7.2)^2^ by using RDP classifier (version 2.2).

### Statistical Analysis

Statistical analysis was carried out using IBM-SPSS V22.0 (IBM, United States). A one-way ANOVA with Duncan’s test was used to determine the significance of physicochemical properties and microbial counts (Li et al., 2020). Principal component analysis (PCA) was performed to analyze the metabolites data by SIMCA-14.1. Paired *t*-test and wilcoxon tests were performed to test the difference in the alpha and beta diversity indices within the stats R package (Version 2.15.3) (Dai et al., 2020). The relationship between microbiota structure and metabolites was analyzed by Two-way Orthogonal Partial Least Squares (O2PLS) and visualized via Cytoscape (v.3.4.0) (Langille et al., 2013). We calculated the Pearson correlation coefficient among the different *Daqu* samples and raw materials to analyze the relationships among them. SourceTracker (version 0.9.8) was used to analyze the sources of microbial communities in *Daqu* (Knights et al., 2011).

## Results

### Physicochemical Properties

The physicochemical properties of inner of fresh *Daqu* (F-I-D), outer of fresh *Daqu* (F-O-D), inner of mature *Daqu* (M-I-D), and outer of mature *Daqu* (M-O-D) were shown in Table 1. The moisture changed with the maturation. Obviously, the moisture in F-*Daqu* was significantly higher (*P* < 0.05) than M-*Daqu*, and the moisture in I-*Daqu* was significantly higher (*P* < 0.05) than O-*Daqu*. The pH of I-*Daqu* decreased slightly while that of O-*Daqu* increased. After the 6 months maturation, both activities of amylase and protease decreased in the I-*Daqu*, while their activities show a opposite trend.

**TABLE 1:** Physicochemical properties of *Daqu* samples.

**Sample**	**Moisture (%)**	**pH**	**Amylase Activity (U/g)**	**Protease Activity (U/g)**
F-I-D	17.28 ± 1.39^d^	6.38 ± 0.05^c^	7.40 ± 0.14^d^	14.80 ± 2.18^b^
F-O-D	12.60 ± 0.35^c^	4.74 ± 0.13^a^	0.73 ± 0.02^a^	1.28 ± 0.34^a^
M-I-D	9.24 ± 0.26^b^	6.29 ± 0.07^c^	4.35 ± 0.03^c^	12.67 ± 1.59^b^
M-O-D	7.63 ± 0.27^a^	5.77 ± 0.08^b^	2.16 ± 0.03^b^	2.87 ± 1.59^a^

*Values represent means ± SD (*n* = 3). Means with different superscripts are significantly different (One-Way ANOVA; *P* < 0.05).*

### Microbiological Analysis

The microbial counts of bacteria and fungi in *Daqu* samples were shown in Table 2. The microbial counts in the F-I-D were significantly higher than that in F-O-D (*P* < 0.05) while the microbial counts of M-I-D was close to that of M-O-D. The difference in microbial counts between I-*Daqu* and O-*Daqu* reduced during maturation. The microbial counts in I-*Daqu* decreased while those in O-*Daqu* increased. The changes of the environment during maturation drove the microbiota inner and outer of *Daqu* to become similar and eventually to form a stable system.

**TABLE 2:** Microbial counts in *Daqu* samples.

**Log_10_ CFU/g**	**F-*Daqu***		**M-*Daqu***	
Microbial groups	F-I-D	F-O-D	M-I-D	M-O-D
Mesophilic aerobic bacteria	8.36 ± 0.11^*d*^	4.73 ± 0.07^*a*^	5.64 ± 0.07^*c*^	5.00 ± 0.04^*b*^
Thermophilic bacteria	6.96 ± 0.07^*d*^	4.63 ± 0.11^*a*^	5.73 ± 0.13^*c*^	5.10 ± 0.06^*b*^
Lactic acid bacteria	8.28 ± 0.02^*d*^	3.64 ± 0.07^*a*^	4.94 ± 0.03^*b*^	5.06 ± 0.07^*c*^
Fungi on RBCA	8.22 ± 0.04^*d*^	2.48 ± 0.01^*a*^	5.17 ± 0.02^*c*^	4.47 ± 0.04^*b*^
Fungi on MEA	8.21 ± 0.02^*d*^	2.49 ± 0.01^*a*^	5.29 ± 0.03^*c*^	4.68 ± 0.03^*b*^

*Values represent means ± SD (*n* = 3). F-I-D: Inner of fresh *Daqu*; F-O-D: Outer of fresh *Daqu*; M-I-D: Inner of mature *Daqu*; M-O-D: Outer of mature *Daqu*. Cluster analysis was based on Unweighted UniFrac distance matrix and the UPGMA method. Means with different superscripts are significantly different (One-Way ANOVA; *P* < 0.05).*

Illumina MiSeq sequencing was utilized to characterize the microbiota structures in F-I-D, F-O-D, M-I-D, and M-O-D. A total of 983,228 high quality reads from V3-V4 region of 16S rRNA gene sequences, and 1,684,859 high quality reads from ITS region were obtained from all samples. For bacteria, there was an average of 49,161 reads per sample, with a range from 29,943 to 73,680 reads. For fungi, there was an average of 84,243 reads per sample, with a range from 32,867 to 144,177 reads. OTUs in samples were defined with a ≥97% sequence identity cutoff, and all samples had high Good’s coverage (1.00). Chao1 richness and other information were shown in Supplementary Table 1. The rarefaction curves of both bacterial and fungal communities approached the saturation plateau, which indicated that the microbial communities were well represented at the sequencing depth (Supplementary Figure 2).

Taxonomic classification of sequences from bacterial communities (a) and fungal communities (b) was showed in Supplementary Figure 3. As for bacterial communities, several types of microbial abundance of *Daqu* such as *Lactobacillus*, *Sphingobacterium*, and *Staphylococcus* decreased after maturation while the abundance of *Kroppenstedtia* and *Lentibacillus* increased, indicating that maturation-related changes had occurred at the genus level (Supplementary Figure 3A). As for fungal communities, the results showed that *Thermoascus*, as the absolute dominant flora, significantly decreased in abundance during the maturation (Supplementary Figure 3B).

**FIGURE 1:**
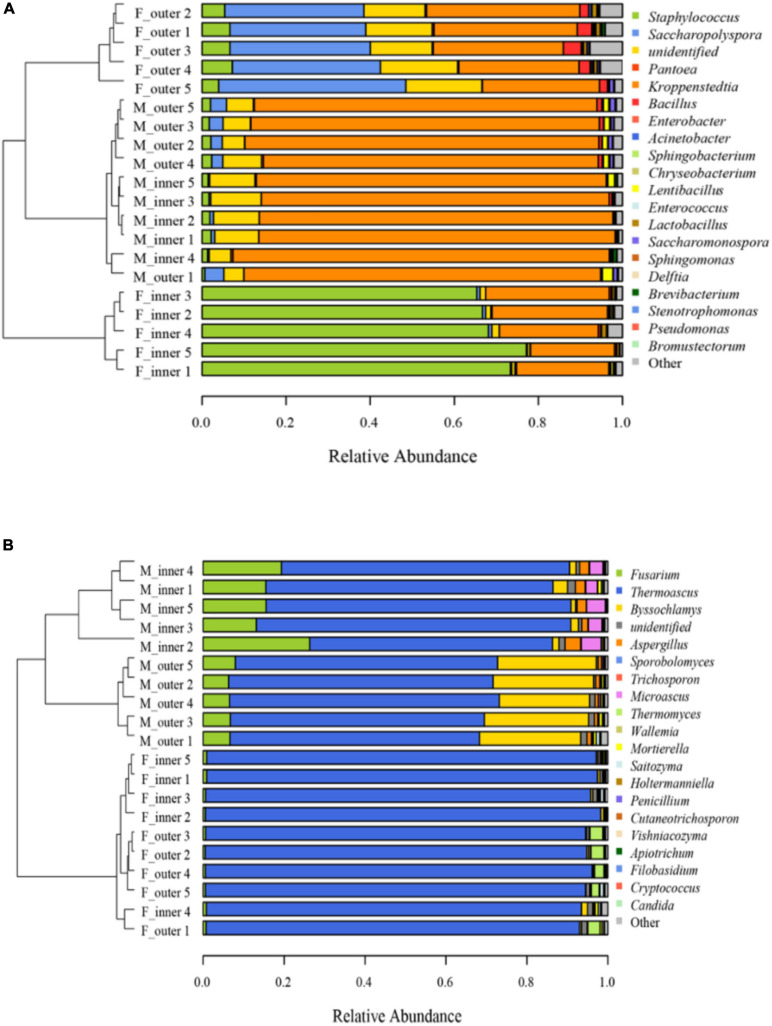
Microbial communities of *Daqu* samples **(A)** Bacterial community. **(B)** Fungal community. F-inner: Inner of fresh *Daqu*; F-outer: Outer of fresh *Daqu*; M-inner: Inner of mature *Daqu*; M-outer: Outer of mature *Daqu*. Cluster analysis was based on Unweighted UniFrac distance matrix and the UPGMA method. Genus level (*n* = 5).

The distribution of microbiota at the genus level was shown in Figure 1. For bacterial communities, *Staphylococcus* predominated in the F-I-D with an average abundance of 70.14%, followed by *Kroppenstedtia* (24.46%). After maturation, *Kroppenstedtia* (84.88%) became the predominant bacterial genus, followed by *Staphylococcus* (1.68%). Besides, *Saccharopolyspora* and *Kroppenstedtia* predominated at the F-O-D with an average abundance of 35.71 and 31.58%, respectively. In M-O-D, *Kroppenstedtia* (82.60%) became the predominant bacterial genus, followed by *Saccharopolyspora* (3.40%). After maturation, the bacterial compositions in both M-I-D and M-O-D became similar (Figure 1A). As for fungal communities (Figure 1B), *Thermoascus* possessed an absolutely dominant position in both F-I-D and F-O-D. After maturation, the proportion of *Fusarium* was increased both in the M-I-D and M-O-D from the fresh *Daqu*. The relative abundance of *Byssochlamys* in M-O-D increased significantly (from 0.27 to 24.46%). Besides, it was mainly the rise of *Microascus* and *Aspergillus* (from 0.03 to 3.80% and from 0.24 to 2.48%, respectively) in M-I-D.

### Multivariate Analysis of Volatile Flavors of *Daqu*

**FIGURE 2:**
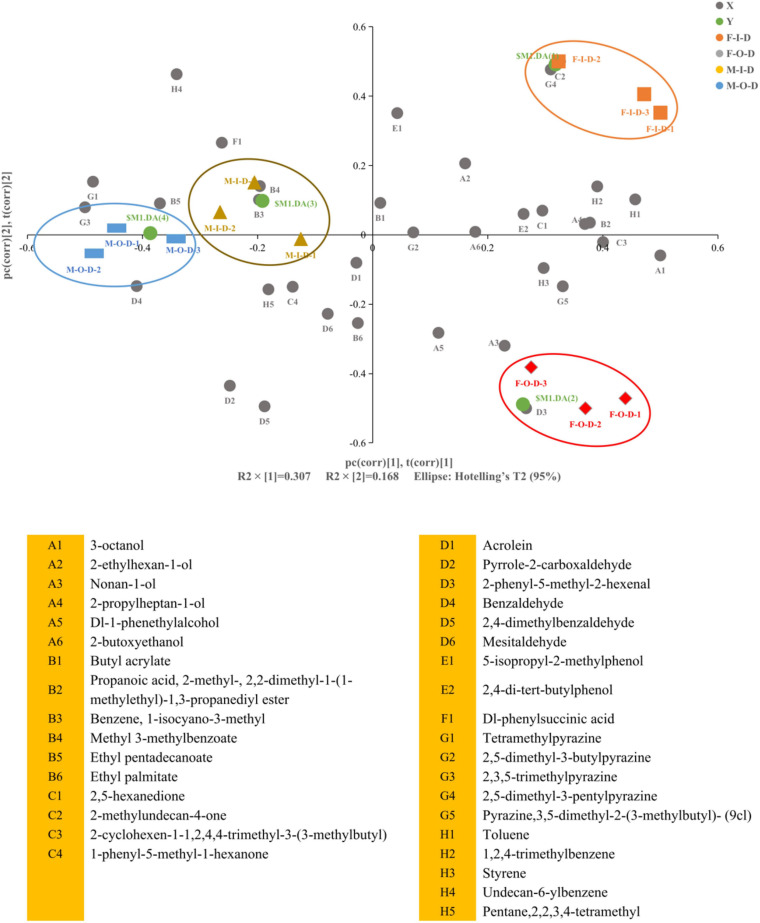
Partial least squares discriminant analysis (PLS-DA) analysis of flavor compounds in *Daqu* samples. F-I-D: Inner of fresh *Daqu*; F-O-D: Outer of fresh *Daqu*; M-I-D: Inner of mature *Daqu*; M-O-D: Outer of mature *Daqu*. X: “
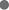
” means flavor compounds. Y: “
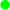
” defines the different classes membership of *Daqu* samples in PLS-DA model. Biplot superimposed the scores and loadings of PLS-DA. The middle number in the sample code represents different plants.

A total of 76 volatile flavors were detected from both F-*Daqu* and M-*Daqu* by HS-SPME-GC-MS, including 11 esters, 15 alcohols, 2 organic acids, 11 ketones, 8 aldehydes, 6 phenols, 5 pyrazine, and 18 others (Supplementary Table 2). Partial least squares discriminant analysis (PLS-DA) was used to analyze the volatile flavor, and the substances with lower variable importance in the projection (VIP) value were eliminated. As shown in Figure 2, the results explained 47.5% of the total variance with R2X (30.7%) and R2Y (16.8%).

The detailed distinctions of volatile flavors profiles in different samples were revealed by the loading plot of PLS-DA. Terms with large VIP were the most relevant for explaining Y. The VIP of benzene, 1-isocyano-3-methyl, 2-phenyl-5-methyl-2-hexenal, 2,4-dimethylbenzaldehyde, 2-methylundecan-4-one, undecan-6-ylbenzene, pyrrole-2-carboxaldehy, 2,5-dimethyl-3-pentylpyrazine, mesitaldehyde, ethyl pentadecanoate, 5-isopropyl-2-methylphenol, dl-1-phenethylalcohol, 2,3,5-trimethylpyrazine, tetramethylpyrazine, toluene, and 3-octanol (VIP > 1.0) contributed to the specificity of *Daqu* samples. Detailed VIP value was provided in the Supplementary Table 3. The categories and the content of volatile flavors decreased after maturation. The main differences between F-I-D and F-O-D were shown in the categories and the content of aldehydes and organic acids (Supplementary Table 2). After maturation, the total number of volatile flavors categories of I-*Daqu* had little change. However, the categories of alcohols, esters and aldehydes increased, while the types of aldehydes and alkanes decreased. It is worth noting that the direct contact between the O-*Daqu* and air resulting in the volatization of some volatile flavors and the decreased abundance of flavor-producing microorganisms (e.g., *Saccharopolyspora*) in O-*Daqu* during the maturation, contributing to the sharp decrease in categories of volatile flavors in O-*Daqu* (Yang et al., 2020).

**FIGURE 3:**
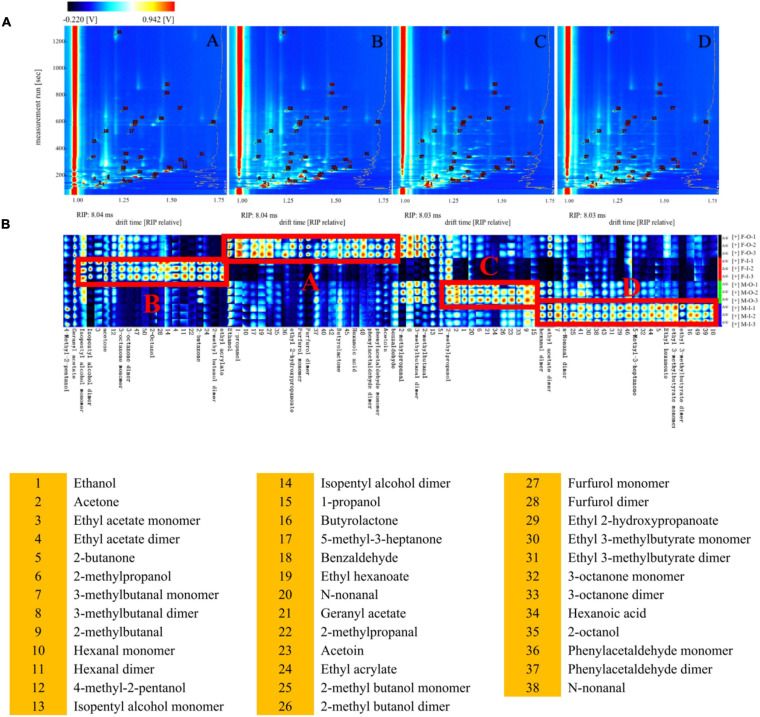
Comparison **(A)** and fingerprints **(B)** of volatile flavors via HS-GC-IMS. A: Inner of fresh *Daqu*; B: Outer of fresh *Daqu*. C: Inner of mature *Daqu*; D: Outer of mature *Daqu*.

As a new flavor detection technology, GC-IMS is widely used for analyzing volatile flavors under the normal atmospheric pressure and highly sensitive to compounds with high electronegativity, which can be used as a supplementary tool for GC-MS to conduct a more comprehensive and systematic detection of the volatile flavors of *Daqu*. The two-dimensional imaging of GC-IMS consisted of drift time, retention time, and the intensity of the ion signals. A total of 88 peaks were detected in *Daqu* samples, and 37 compounds, including 14 aldehydes, 7 alcohols, 6 ketones, 1 organic acid, and 9 esters, were identified by the NIST 2014 and IMS database (Supplementary Table 4). Many volatile flavors present in different degree of polymerization, such as monomers and dimers depending upon their concentrations. Figure 3A displayed that these products pass through the drift region and multiple signals can be observed as a single compound due to the formation of adducts between the ions and neutral molecules (such as dimers and trimers), including 2-methyl butanol, 2-methylbutanal, 3-methylbutanal, 3-octanone, ethyl 2-hydroxypropanoate, ethyl acrylate, furfurol dimer, hexanal, isopentyl alcohol, n-non-anal, and phenylacetaldehyde. These products exhibited similar retention times, but different drift time (Fan et al., 2021). Figure 3B displays that all compounds, identified via GC-IMS, have been selected to compare the differences of four types of *Daqu* samples. Each column represented the signal peak of one volatile compound. The brighter spot indicated the higher concentration of the volatile compound. The monomers and dimers of the same compound were indicated by different columns with the same compound name. However, due to the proton affinity and higher concentration, the drift time of dimers increased and showed distinct spots in the fingerprint (Yang et al., 2020). The total volatile flavors of F-O-D and M-O-D were relatively similar, and the same condition was shown in M-O-D and M-I-D based on the spot color. Compounds of regional A in Figure 3B (ethanol, ethyl 2-hydroxy propyl alcohol, propionic acid, furfural, butyrolactone, caproic acid, benzene acetaldehyde, ethyl benzoin, benzene, and formaldehyde, etc.), regional B (isoamyl alcohol, acetone, ketone, octanol, butanone, 2-methyl ethyl acrylate, and butyl alcohol), regional C (2-methyl propyl alcohol, etc.), and regional D (hexanal, ethyl acetate, nonyl aldehyde and 3-methyl ethyl caproate, ethyl butyrate, etc.) represented the characteristics of volatile flavors in F-O-D, F-I-D, M-O-D, and M-I-D.

### Polar Metabolites of *Daqu*

Nuclear magnetic resonance (^1^H NMR spectroscopy) coupled with multivariate statistical analysis was used to investigate the differences of the *Daqu* samples in the polar metabolic changes to better characterize *Daqu* maturation. As shown in Supplementary Table 5, a total of 76 polar metabolites including 17 esters, 19 amino acids and their derivatives, 6 alkalamides, 4 alcohols, 30 sugars and their derivatives, and salt of organic acid were detected in *Daqu* samples. Due to a series of complicated metabolism like the Maillard reaction and enzymatic reaction, the polar metabolites in *Daqu* increased with the maturation. For example, in terms of the type and content of esters, F-I-D and F-O-D differed greatly, while M-I-D and M-O-D became closer at the end of maturation. These trends were caused by the shifts of microbiota during maturation. Of all the polar metabolites, the most abundant compounds were alcohols, especially ethanol and glycerol. They were the substrates or precursors for a series of subsequent reactions (Zheng et al., 2011). The polar metabolites’ contents especially esters, amino acids and sugars in F-I-D were significantly lower than those in F-O-D.

### Network Analysis of the Interactions Among Microbiota, Polar Metabolites and Volatile Flavors in *Daqu*

**FIGURE 4:**
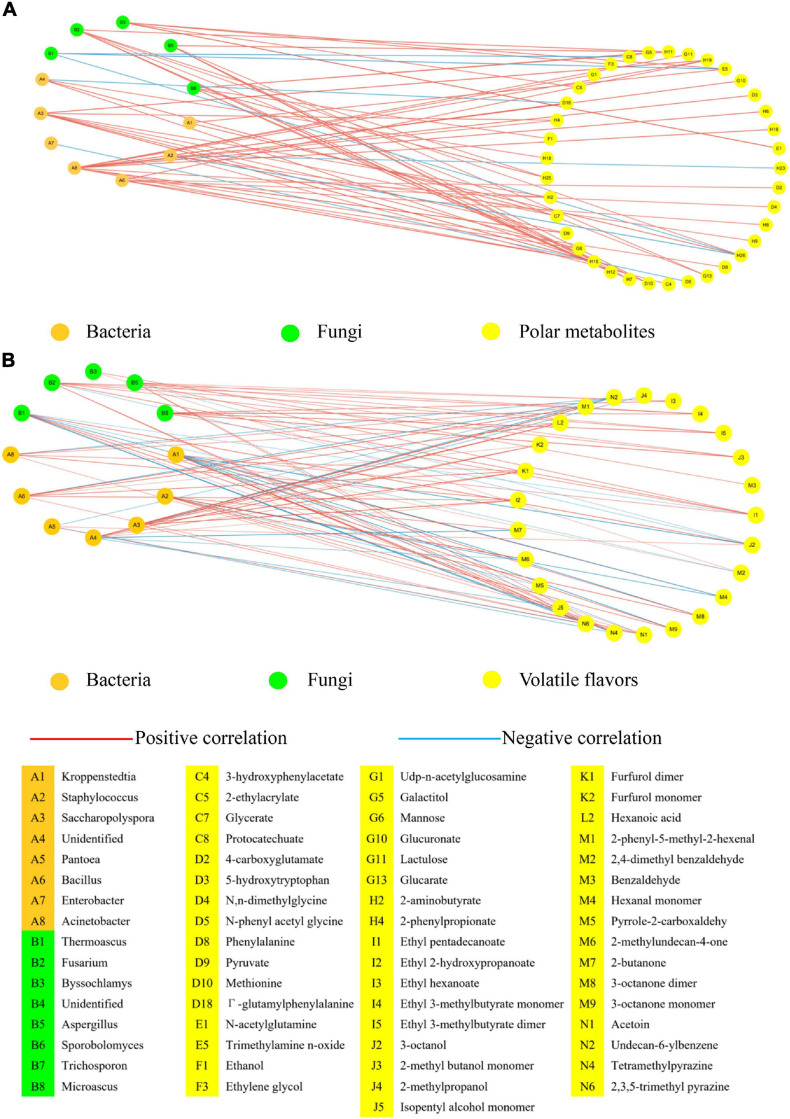
Network analysis of the interactions among microbiota, polar metabolites (^1^H NMR) and volatile flavors (HS-SPME-GC-MS and HS-GC-IMS). **(A)** The correlated network between microbiota and polar metabolites. **(B)** The correlated network between microbiota and volatile flavors. Population data was considered at the OTU level and statistically significant Pearson correlations were calculated among *Daqu* samples. A connection stands for a significant (*P* < 0.05) and positive (Pearson correlation > |0.7|) correlation.

We analyzed the correlations among microbiota, polar metabolites, and volatile flavors in *Daqu* via Pearson correlation analysis (Figure 4) and the detailed value of Pearson correlation coefficients (Pearson correlation > |0.7|) was shown in Supplementary Table 6. Correlation analysis between microbiota and polar metabolites was shown in Figure 4A. Bacteria such as *Saccharopolyspora*, *Bacillus*, and *Acinetobacter* showed a strong correlation with polar metabolites. *Saccharopolyspora* had the highest abundance in F-O-D, which produced more polar metabolites such as esters, amino acids and their derivatives, and sugars and their derivatives in the production process of *Daqu*. For fungi, the correlation network showed that *Thermoascus* was negatively correlated with phenylalanine, trimethylamine n-oxide, and isovalerate. In previous studies, *Thermoascus* could produce some thermostable enzyme and epipolythiodioxopiperazine metabolite derived from the amino acid pathway, and these metabolites exhibited antibacterial and antiviral properties (Leite et al., 2008; Siddiquee, 2018). The abundances of *Fusarium*, *Byssochlamys*, *Aspergillus*, and *Microascus* in M-*Daqu* were significantly higher than those in F-*Daqu* and they showed a strong correlation with 23 polar metabolites including amino acids and their derivatives and esters such as 3-hydroxyisovalerate, propionate, fumarate, acetoacetate, and methionine. These fungi were closely related to the synthesis of most esters and some amino acids during maturation.

**FIGURE 5:**
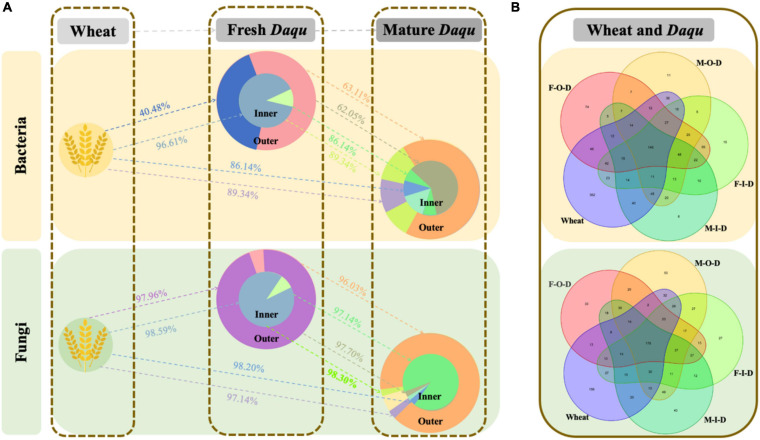
SourceTracker results for the wheat and *Daqu* sample via SourceTracker. **(A)** Estimation of shift pathway and proportion. **(B)** Specific numbers of OTUs. The “→” represent microbiota shift direction. F-I-D: Inner of fresh *Daqu*; F-O-D: Outer of fresh *Daqu*; M-I-D: Inner of mature *Daqu*; M-O-D: Outer of mature *Daqu*.

Correlation analysis between microbiota and volatile flavors showed that the abundance of *Kroppenstedtia* was positively correlated with tetramethylpyrazine, 2,3,5-trimethyl pyrazine, 2,4-dimethyl benzaldehyde, hexanal monomer, and ethyl pentadecanoate (Figure 5B). The abundance of *Kroppenstedtia* increased significantly after *Daqu* maturation, and the content of associated pyrazines such as tetramethylpyrazine and 2,3,5-trimethyl pyrazine were also significantly higher in mature *Daqu* than those in fresh *Daqu*. Interestingly, *Kroppenstedtia*’s abundance was negatively correlated with 3-octanone and 3-octanol, while *Thermoascus*’s abundance was positively correlated with these substances. From the point of view of the microbiota succession, *Kroppenstedtia* and *Thermoascus* showed the rule of ebb and flow because *Kroppenstedtia* had a higher tolerance to ethanol which was accumulated in the later step of *Daqu* maturation. *Bacillus* has a positive correlation with esters, sugars and derivatives, organic acids, aldehydes and ketones due to its high-temperature resistance and strong secretion enzyme function (Siddiquee, 2018). *Fusarium* and *Aspergillus* had a similar correlation with volatile flavors, and both had a strong positive correlation with esters and alcohols. Studies have pointed out that *Fusarium* could decompose cellulose and other organic compounds and could degrade phenolic compounds and polycyclic aromatic hydrocarbons at the same time (Leite et al., 2008). *Aspergillus* could decompose proteins to produce volatile flavors and also produce organic acids (Yu et al., 2021).

*Kroppenstedtia* was positively correlated with some pyrazines such as tetramethylpyrazine and 2,3,5-trimethyl pyrazine. Other bacteria such as *Saccharopolyspora*, *Bacillus*, and *Acinetobacter* were positively correlated with organic acid, amino acids and their derivatives, and sugars and their derivatives. Besides, *Fusarium* and *Aspergillus* had a strong positive correlation with esters, alcohols, organic acid while *Thermoascus* showed a negative correlation with phenylalanine, trimethylamine n-oxide, and isovalerate.

### SourceTracker Analysis of Raw Materials and *Daqu*

SourceTracker has been widely used as an important tool of tracking microbial source in many fields, including food fermentation and meat processing (Du et al., 2019). Thus, we exploited SourceTracker to estimate the source and succession of microbiota in *Daqu*. In addition to the microbiota in *Daqu*, we also analyzed the microbiota on the surface of wheat, the raw material for *Daqu*-making (Supplementary Table 7). The traceability of microbiota in high-temperature *Daqu* were shown in Figure 5. Schematic diagram showed that the microbiota shifted from wheat to fresh *Daqu* and finally to mature *Daqu*. In particular, the proportion of microbiota shifted as shown in Figure 5A and the specific number of OTUs were shown in Figure 5B. The bacterial community of F-I-D mainly came from wheat (96.61%), while only about 40.48% of F-O-D came from wheat. In detail, *Staphylococcus*, *Saccharopolyspora*, and *Kroppenstedtia* might shifted to *Daqu* from wheat and became dominant bacteria in *Daqu*. Besides, most of the wheat-based microbiota were shown in the I-*Daqu* (Du et al., 2019). Compared with F-*Daqu*, M-*Daqu* were more from the F-I-D rather than the F-O-D. Approximately 89.34% of bacterial community in M-O-D was shifted from F-I-D, slightly higher than that in M-I-D (86.14).

Combined with the results of Illumina MiSeq sequencing, the fungal traceability of I-*Duqu* and O-*Daqu* was similar. However, the difference of fungal community (Figure 1B) in M-I-D and M-O-D after maturation became larger than that of the maturation at the beginning. *Thermoascus*, the dominant fungus with the highest abundance in the *Daqu* maturation, was also detected on the surface of wheat. It could be inferred that part of *Thermoascus* shifted from wheat to the F-*Daqu* and then its abundance reduced due to the growth of thermophilic bacteria and *Byssochlamys* (Du et al., 2019). There were also some fungi such as *Fusarium* and *Aspergillus* in wheat and M-*Daqu* with high abundance, but low abundance in F-*Daqu*.

## Discussion

This study explored the microbiota from wheat and the contribution of the inner and outer of *Daqu* microbiota during maturation. Physicochemical properties, including moisture, pH, amylase activity, and protease activity reflect the maturity and quality of *Daqu* (Deng et al., 2020). The moisture in F-*Daqu* was higher than that in M-*Daqu*, and the moisture in I-*Daqu* was higher than that of O-*Daqu*. It is due to the open and well-ventilated storage condition and the dry and cool air in the environment, which lead more moisture loss in O-*Daqu* (Chen et al., 2021). The difference in pH is related to the difference in composition of organic acids and the changes in the diversity of slightly acidic bacteria like the *Acetobacter* and *Lactobacillus* (Fan et al., 2019). The changes in the microbiota that produced amylase and protease might be the mainly reason for the changes in enzyme activities (Guan et al., 2020).

During the maturation, the microbiota in different parts of *Daqu* underwent great changes. The bacterial communities of F-I-D and F-O-D were quite different because microbes from the environment had a great influence on F-O-D exposed to the air during the whole production process, while most of the microbiome in F-I-D came from raw materials (He et al., 2019). Besides, the bacterial community of M-I-D and M-O-D became similar after maturation. Compared with medium-temperature and low-temperature *Daqu*, high-temperature *Daqu* had relatively fewer bacterial species due to high fermentation temperature (Zheng et al., 2011). The abundance of *Saccharopolyspora* was high in F-O-D, and the microbiota came from the environment or the raw materials that provided the initial microbiota for enzymatic reactions to produce metabolites. In the mature step of *Daqu*, the microbial abundance in *Daqu* decreased after high temperature fermentation, mainly including some thermotolerant and thermophilic bacteria. *Kroppenstedtia* became the absolute dominant strain in the M-*Daqu*. It was isolated recently and was identified in several types of *Daqu* (Fan et al., 2019). However, its role in *Daqu* manufacture is not clear. Studies had shown that *Lactobacillus* could inhibit *Kroppenstedtia* to a certain extent, which explained that the decline of *Lactobacillus* abundance promoted the growth of *Kroppenstedtia* after maturation (Yan et al., 2016). For *Staphylococcus*, the absolute dominant bacterial genera in F-I-D, Stevens et al. (2015) discovered that *Staphylococcus* were dominant across different foot sites and comprised almost the entire bacterial population on the plantar surface (Stevens et al., 2015). Du et al. (2019) proved that both the raw materials and the environments acted as important sources for *Daqu* microbiota (Du et al., 2019). Because of the relatively open processing environment and the barefoot stepping process by women, there might be some microbiota such as *Staphylococcus* originating from the environment or women’ feet in *Daqu* (Wang et al., 2021). During maturation, it was gradually inhibited by *Kroppenstedtia* (He et al., 2019). For fungal communities, few fungi can stand the temperature in the high-temperature *Daqu* making process because they prefer the low temperature environment for reproduction. However, the microflora showed a tendency of enrichment after maturation. Specifically, the abundance of *Fusarium*, *Byssochlamys*, *Aspergillus*, and *Microascus* in mature *Daqu* was significantly higher than that in fresh *Daqu*. *Fusarium* was widely found in nature especially in crops such as wheat and is beneficial to agriculture such as gibberellin (Franco et al., 2021). *Fusarium* in fresh *Daqu* was mainly derived from wheat and had a certain inhibitory effect on *Thermoascus* in the maturation process and became one of the dominant strains in mature *Daqu* (He et al., 2019). As the absolute dominant fungus in fresh *Daqu*, *Thermoascus* was negatively correlated with ethanol, and the accumulation of ethanol in the maturation process had a certain inhibitory effect on it (Wei et al., 2021).

The maturation of *Daqu* involves the changes of temperature, and the competition between bacteria and fungi lead to the change of volatile flavors produced by their metabolisms, which is also the reason for the production of the abundant volatile flavors in *Daqu* (Chen et al., 2021). From all volatile flavors detected by HS-SPME-GC-MS and HS-GC-IMS, there were obvious differences among the four *Daqu* samples. The incubation temperature of sauce-flavor *Daqu* is usually very high (reaching up to 70°C), and the Maillard reaction causes color changes and produces a series of volatile flavors like ketones, aldehydes, and heterocyclic compounds (Zheng et al., 2011). Besides, Sauce-flavor *Daqu* bricks are big and heavy (above 4.8 kg), which results in the different temperatures of the I-*Daqu* and O-*Daqu* and color gradients from brown inside to yellow outside (Huang et al., 2020). According to the detected results, F-I-D and F-O-D show great differences in compound compositions because of the metabolic reactions at different temperatures before maturation. At the end of maturation, the compositions of M-I-D and M-O-D became similar. It is probably because microbial succession, and compounds exchange took place in the inner and outer *Daqu*, leading to the formation of a relatively uniform system during maturation. Longitudinally, microbiota succession had been in progress in the whole maturation period. The bacterial community structures of F-*Daqu* and M-*Daqu* were very different, mainly reflected in the abundance of *Kroppenstedtia*, *Saccharopolyspora*, *Staphylococcus*, and *Bacillus*. High-intensity *Staphylococcus* produced volatile flavors such as 3-methyl-1-butanol, 2-butanone, and acetoin, which might play important roles in *Baijiu* production (Guan et al., 2020). *Bacillus* could convert starch and proteins into glucose and amino acids by secreting amylase, protease, cellulases, glucanases, and other enzymes, thereby contributing to the development of volatile flavors (Fan G. S. et al., 2020). At the same time, F-*Daqu* and M-*Daqu* showed significant differences in fungal community structure. After maturation, the abundance of *Fusarium* and *Byssochlamys* increased significantly, and the interaction between microorganisms became more complex. This played a decisive role in the change of metabolites between F-*Daqu* and M-*Daqu*. Besides, ^1^H NMR spectroscopy was used to investigate the differences of the *Daqu* samples in the polar metabolic changes. The results show that esters, amino acids and sugars in F-I-D were significantly lower than those in F-O-D. This was probably because the abundance of *Thermomyces* in F-O-D was significantly higher than that in F-I-D. *Thermomyces* secreted cellulase, amylase, and protease, which converted starch, cellulose, and other raw substances into small molecules such as glucose and amino acids. These small molecules supplied nutrition and energy for the growth and metabolism of the microbial community (Ali et al., 2019). The contents of esters in M-I-D and M-O-D were similar because of the similar ester producing bacterial community structure between M-I-D and M-O-D, while the higher abundance of *Aspergillus* in M-I-D contributed to the significantly higher contents of amino acids and derivatives, sugars and their derivatives in M-I-D than M-O-D due to *Aspergillus*’s ability to decompose the large molecules such as starch into small polar metabolites (e.g., glucose) (Ali et al., 2019).

Some studies in medium temperature *Daqu* had shown that fungal communities were mainly originated from *Daqu* making environments (especially tools and indoor ground) while most of bacterial communities were from raw materials (Du et al., 2019). During the maturation, the bacterial community of wheat participated in microbiota succession and showed a migration phenomenon from inner to outer of *Daqu*. In other words, I-*Daqu* was an important channel for wheat microorganisms to act on *Daqu* (from wheat to F-I-D to the whole *Daqu*). Some bacteria migrated to the O-*Daqu* from I-*Daqu*, mainly *Kroppenstedtia*, *Saccharopolyspora*, and *Staphylococcus*, etc., which resulted in a similar and stable community structure in M-I-D and M-O-D. In short, the core bacterial community in the F-I-D was the most important driving force for the maturation of *Daqu*. Wheat might be the source of these fungi whose growth was inhibited during the *Daqu* making (Yu et al., 2021). Another important reason was that these fungi in the environment transferred to the outer of *Daqu* and enriched in the maturation process and reproduced during maturation under suitable moisture and temperature, and finally increased its abundance in mature *Daqu* (Du et al., 2019). On this basis, the mechanism of raw material microbiota in *Daqu* still needs to be further studied.

## Conclusion

Taken together, wheat-originated microbes were an important source for the microbial community formation of *Daqu*, especially the inner of *Daqu*. The bacterial community in the inner of *Daqu* was an important driving force of maturation. During the maturation, the difference in the bacterial community between the inner and outer of *Daqu* decreased and the predominant microbes shifted from *Saccharopolyspora* (outer) and *Staphylococcus* (inner) of fresh *Daqu* to *Kroppenstedtia* (both outer and inner) of mature *Daqu*, in which was indicated by the difference in metabolites. For fungal community, the predominant fungi shifted from *Thermoascus* (both outer and inner) in fresh *Daqu* to *Byssochlamys* (outer) and *Fusarium* (inner) in mature *Daqu*. Bacteria such as *Saccharopolyspora*, *Bacillus*, and *Acinetobacter* had a strong correlation with the contents of esters, amino acids and their derivatives, and sugars and their derivatives, which confirmed the important role of bacterial community in the inner of *Daqu*. Our study provided a deeper understanding of the role of microbiota during *Daqu* maturation and improvement of the quality and stability of *Daqu*.

## Data Availability Statement

The data presented in the study are deposited in the NCBI Sequence Read Archive (SRA) repository, accession number (PRJNA727444).

## Author Contributions

YZ designed and conducted the experimental work assisted by XC, performed the data analysis, and wrote the first draft of the manuscript. YX contributed to the manuscript revision. B-ZH contributed to the supervision, manuscript revision, and overall support of this study. All authors read and approved the final version of the manuscript.

## Conflict of Interest

YS, WC, and XW were all employed by Sichuan Langjiu Co., Ltd. The remaining authors declare that the research was conducted in the absence of any commercial or financial relationships that could be construed as a potential conflict of interest.

## Footnotes

1 http://www.arb-silva.de

2 http://unite.ut.ee/index.php
