# Supplementary material for: Understanding the Shifts of Microbial Community and Metabolite Profile From Wheat to Mature Daqu
Source: Front Microbiol. 2021 Jul 12;12:714726. doi: 10.3389/fmicb.2021.714726 (PMC8312246; doi:10.3389/fmicb.2021.714726)
Supplement: Supplementary file 1 [file Data_Sheet_1.doc]

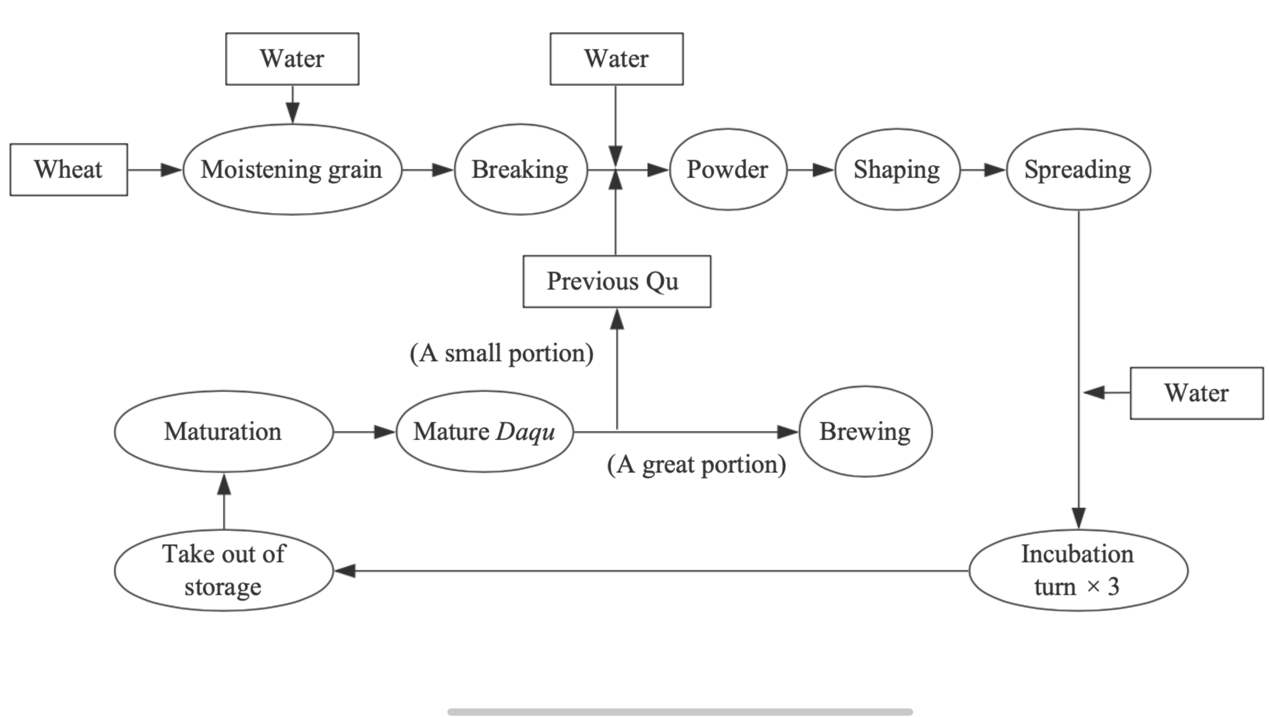


**Fig. S1** The production process of Sauce-flavor *Daqu*.

**Table S1 Summary of sequencing results of *Daqu* samples.**

| Type | Sample ID | Reads | OTUs^a^ | Chao1 | Good’s coverage |
| --- | --- | --- | --- | --- | --- |
| Bacteria | F-I-D-1 | 43088 | 309 | 321.58 | 1.00 |
|  | F-I-D-2 | 59683 | 281 | 390.68 | 1.00 |
|  | F-I-D-3 | 29943 | 339 | 305.15 | 1.00 |
|  | F-I-D-4 | 55375 | 290 | 397.12 | 1.00 |
|  | F-I-D-5 | 48996 | 266 | 201.88 | 1.00 |
|  | F-O-D-1 | 73680 | 157 | 393.65 | 1.00 |
|  | F-O-D-2 | 39466 | 237 | 393.49 | 1.00 |
|  | F-O-D-3 | 59840 | 171 | 399.38 | 1.00 |
|  | F-O-D-4 | 53013 | 213 | 390.74 | 1.00 |
|  | F-O-D-5 | 47610 | 194 | 361.01 | 1.00 |
|  | M-I-D-1 | 44977 | 209 | 210.03 | 1.00 |
|  | M-I-D-2 | 41973 | 234 | 261.33 | 1.00 |
|  | M-I-D-3 | 59319 | 216 | 271.00 | 1.00 |
|  | M-I-D-4 | 45740 | 290 | 457.81 | 1.00 |
|  | M-I-D-5 | 46738 | 119 | 315.32 | 1.00 |
|  | M-O-D-1 | 57826 | 147 | 293.50 | 1.00 |
|  | M-O-D-2 | 34162 | 164 | 370.90 | 1.00 |
|  | M-O-D-3 | 69789 | 156 | 288.30 | 1.00 |
|  | M-O-D-4 | 31739 | 211 | 363.72 | 1.00 |
|  | M-O-D-5 | 40271 | 189 | 383.50 | 1.00 |
| Fungi | F-I-D-1 | 88577 | 218 | 238.61 | 1.00 |
|  | F-I-D-2 | 32867 | 166 | 182.90 | 1.00 |
|  | F-I-D-3 | 90645 | 197 | 375.50 | 1.00 |
|  | F-I-D-4 | 87456 | 152 | 517.07 | 1.00 |
|  | F-I-D-5 | 106718 | 232 | 337.18 | 1.00 |
|  | F-O-D-1 | 144177 | 315 | 374.09 | 1.00 |
|  | F-O-D-2 | 133738 | 198 | 261.00 | 1.00 |
|  | F-O-D-3 | 111146 | 234 | 318.83 | 1.00 |
|  | F-O-D-4 | 36531 | 209 | 298.30 | 1.00 |
|  | F-O-D-5 | 112487 | 172 | 407.78 | 1.00 |
|  | M-I-D-1 | 54305 | 162 | 377.11 | 1.00 |
|  | M-I-D-2 | 101065 | 114 | 341.83 | 1.00 |
|  | M-I-D-3 | 64053 | 224 | 291.07 | 1.00 |
|  | M-I-D-4 | 73580 | 292 | 343.52 | 1.00 |
|  | M-I-D-5 | 37098 | 199 | 350.33 | 1.00 |
|  | M-O-D-1 | 94765 | 278 | 587.56 | 1.00 |
|  | M-O-D-2 | 108432 | 223 | 313.18 | 1.00 |
|  | M-O-D-3 | 76328 | 212 | 375.97 | 1.00 |
|  | M-O-D-4 | 113409 | 196 | 381.44 | 1.00 |
|  | M-O-D-5 | 106059 | 152 | 292.00 | 1.00 |

^a^ Operational taxonomic units (OTUs) were defined with 97% similarity level.

F-I-D: Inner of fresh *Daqu*; F-O-D: Outer of fresh *Daqu*; M-I-D: Inner of mature *Daqu*; F-O-D: Outer of mature *Daqu*.


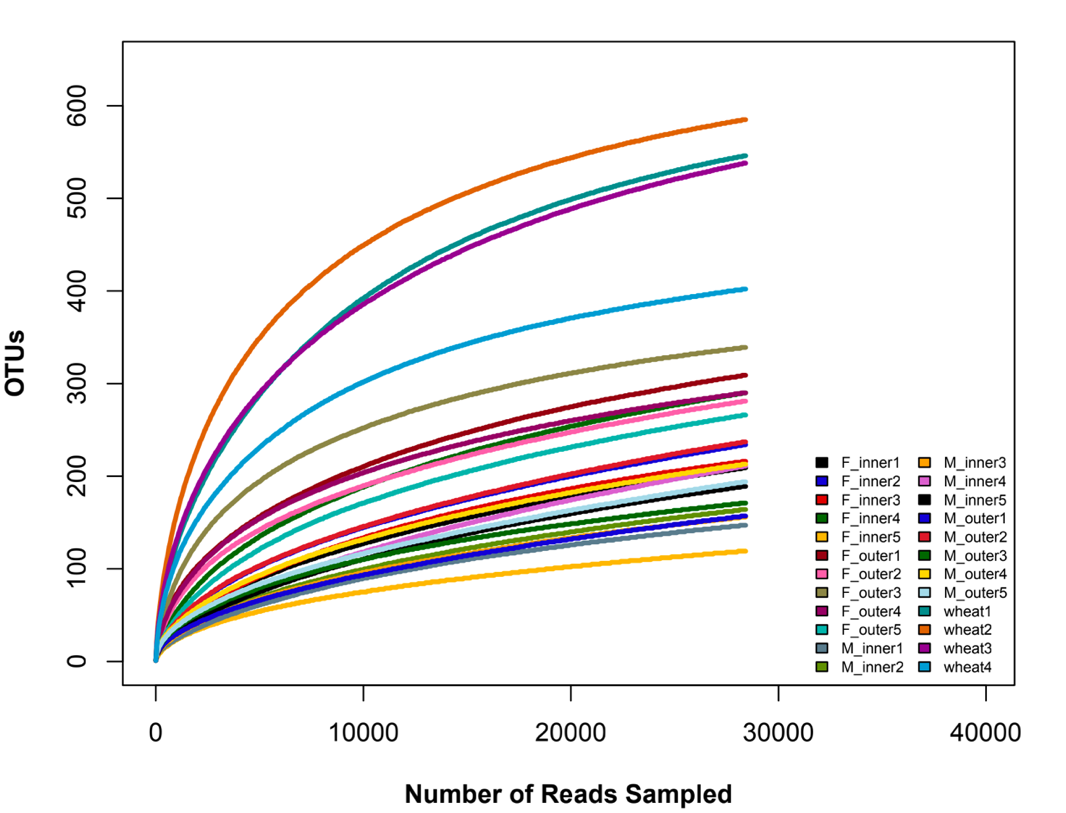


**Fig. S2** The rarefaction curves of both bacterial and fungal. (F-inner: inner of fresh *Daqu*; F-outer: outer of fresh *Daqu*; M-inner: inner of mature *Daqu*; M-outer: outer of mature *Daqu*)


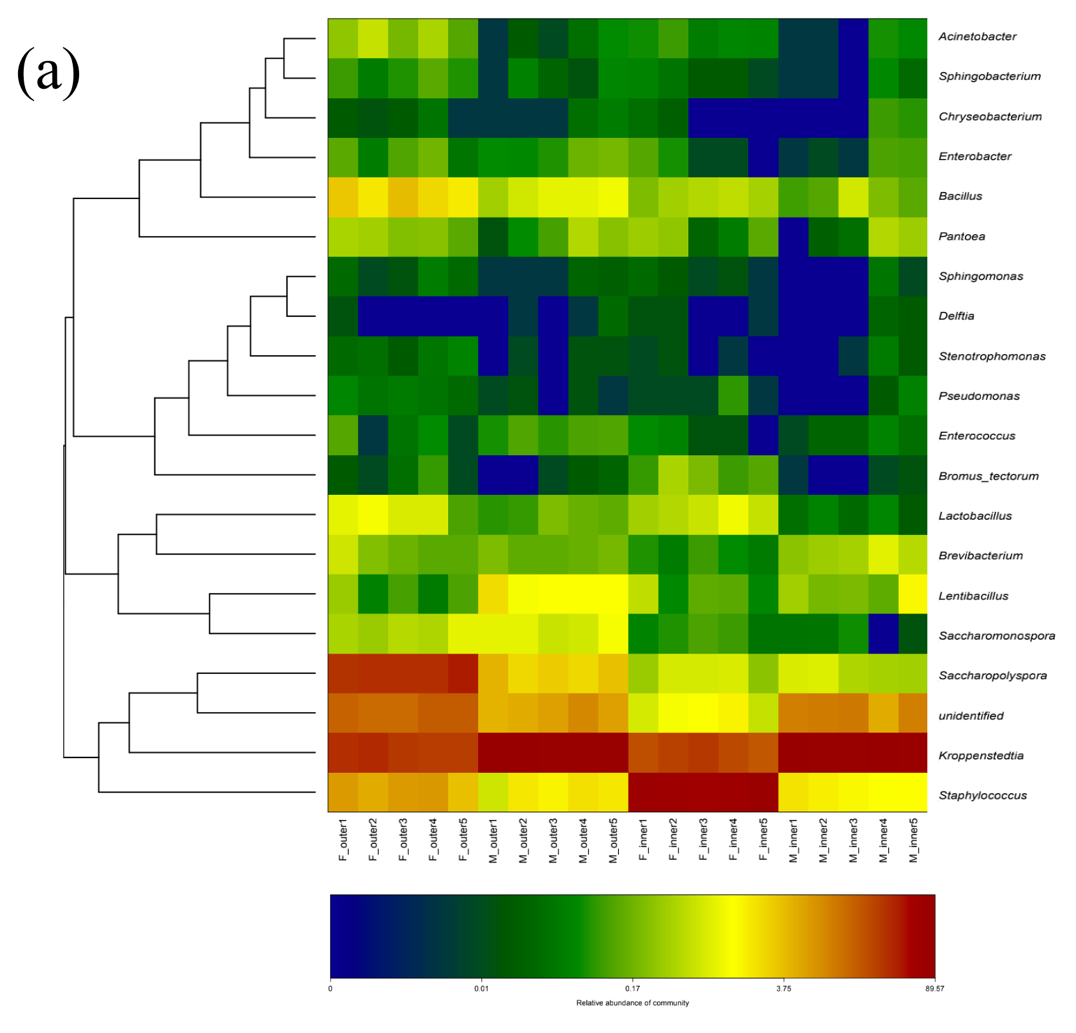


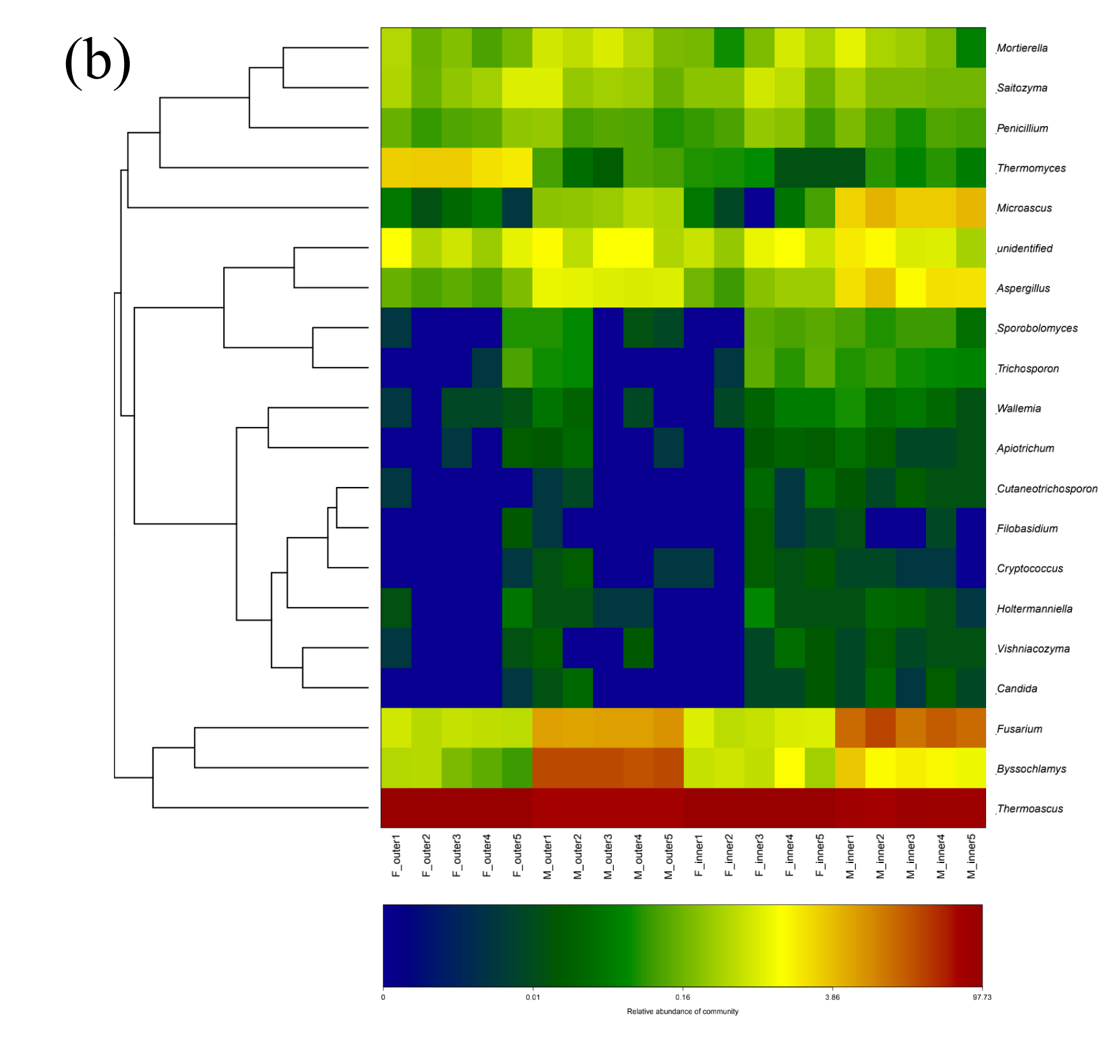


**Fig. S3** Taxonomic classification of sequences from bacterial communities (a) and fungal communities (b) of samples (F-inner, inner of fresh *Daqu*; F-outer, outer of fresh *Daqu*; M-inner, inner of mature *Daqu*; M-outer, outer of mature *Daqu*) at genus level. The top 20 genera detected in *Daqu* were shown. The relative abundance of each genus was indicated by color intensity in a heat map (n=5).

**Table S2** The volatile flavors of different *Daqu* samples by HS-SPME-GC-MS.

| Flavors^a^ | No. | Compounds | CAS | Formula | MW |
| --- | --- | --- | --- | --- | --- |
| Alcohols | 1 | 3-octanol | 589-98-0 | C_8_H_18_O | 130.23 |
|  | 2 | 2-ethylhexan-1-ol | 104-76-7 | C_8_H_18_O | 130.23 |
|  | 3 | Nonan-1-ol | 143-08-8 | C_9_H_20_O | 144.25 |
|  | 4 | 2-propylheptan-1-ol | 10042-59-8 | C_10_H_22_O | 158.28 |
|  | 5 | Decyl alcohol | 112-30-1 | C_10_H_22_O | 158.28 |
|  | 6 | Dl-1-phenethylalcohol | 98-85-1 | C_8_H_10_O | 122.17 |
|  | 7 | (s, s)-2,3-butanediol | 19132-06-0 | C_4_H_10_O_2_ | 90.12 |
|  | 8 | 1-heptanol, 5-methyl-, (5s) | 57803-73-3 | C_8_H_18_O | 130.23 |
|  | 9 | Phenethyl alcohol | 60-12-8 | C_8_H_10_O | 122.16 |
|  | 10 | 1-octanol, 6-methyl-, (6s) | 110453-78-6 | C_9_H_20_O | 144.25 |
|  | 11 | 1-phenyl-2-propanol | 14898-87-4 | C_9_H_12_O | 136.19 |
|  | 12 | Tetrahydrolavandulol | 2051-33-4 | C_10_H_22_O | 158.28 |
|  | 13 | 2-methyl-1-octanol | 818-81-5 | C_9_H_20_O | 144.25 |
|  | 14 | 2-phenoxyethanol | 122-99-6 | C_8_H_10_O_2_ | 138.16 |
|  | 15 | 2-butoxyethanol | 111-76-2 | C_6_H_14_O_2_ | 118.17 |
| Esters | 16 | Butyl acrylate | 141-32-2 | C_7_H_12_O_2_ | 128.17 |
|  | 17 | propanoic acid, 2-methyl-, 2,2-dimethyl-1-(1-methylethyl)-1,3-propanediyl ester | 6846-50-0 | C_16_H_30_O_4_ | 286.41 |
|  | 18 | Metolcarb | 1129-41-5 | C_9_H_11_NO_2_ | 165.19 |
|  | 19 | Benzene, 1-isocyano-3-methyl | 20600-54-8 | C_8_H_7_N | 117.15 |
|  | 20 | Ethyl myristate | 124-06-1 | C_16_H_32_O_2_ | 256.42 |
|  | 21 | Methyl 3-methylbenzoate | 99-36-5 | C_9_H_10_O_2_ | 150.17 |
|  | 22 | Ethyl acetate | 141-78-6 | C_4_H_8_O_2_ | 88.11 |
|  | 23 | Ethyl pentadecanoate | 41114-00-5 | C_17_H_34_O_2_ | 270.45 |
|  | 24 | Methyl 2-methylhexadecanoate | 2490-53-1 | C_18_H_36_O_2_ | 284.48 |
|  | 25 | Ethyl palmitate | 628-97-7 | C_18_H_36_O_2_ | 284.48 |
|  | 26 | Butyl isobutyrate | 97-87-0 | C_8_H_16_O_2_ | 144.21 |
| Ketones | 27 | 2,5-hexanedione | 110-13-4 | C_6_H_10_O_2_ | 114.14 |
|  | 28 | Acetoin | 513-86-0 | C_4_H_8_O_2_ | 88.11 |
|  | 29 | 2-methylundecan-4-one | 19594-40-2 | C_12_H_24_O | 184.32 |
|  | 30 | 3-tert-butyl-2-pyrazolin-5-one | 29211-68-5 | C_7_H_12_N_2_O | 140.18 |
|  | 31 | 2-acetylpyrrole | 1072-83-9 | C_6_H_7_NO | 109.13 |
|  | 32 | 3,6-dimethyl-4h-furo<3,2-c>pyran-4-on | 36745-38-7 | C_9_H_8_O_3_ | 164.16 |
|  | 33 | 2-cyclohexen-1-1,2,4,4-trimethyl-3-(3-methylbutyl) | 88725-82-0 | C_14_H_24_O | 208.34 |
|  | 34 | 2,2,5-trimethylhexane-3,4-dione | 20633-03-8 | C_9_H_16_O_2_ | 156.22 |
|  | 35 | 3-octanone | 106-68-3 | C_8_H_16_O | 128.21 |
|  | 36 | 3-ethylacetophenone | 22699-70-3 | C_10_H_12_O | 148.20 |
|  | 37 | 1-phenyl-5-methyl-1-hexanone | 25552-17-4 | C_13_H_18_O | 190.28 |
| Aldehyde | 38 | Acrolein | 107-02-8 | C_3_H_4_O | 56.06 |
|  | 39 | 3,5,5-trimethyl-hexanal | 5435-64-3 | C_9_H_18_O | 142.24 |
|  | 40 | M-phthalaldehyde | 626-19-7 | C_8_H_6_O_2_ | 134.13 |
|  | 41 | Pyrrole-2-carboxaldehyde | 1003-29-8 | C_5_H_5_NO | 95.10 |
|  | 42 | 2-phenyl-5-methyl-2-hexenal | 21834-92-4 | C_13_H_16_O | 188.27 |
|  | 43 | Benzaldehyde | 100-52-7 | C_7_H_6_O | 106.12 |
|  | 44 | 2,4-dimethylbenzaldehyde | 15764-16-6 | C_9_H_10_O | 134.18 |
|  | 45 | Mesitaldehyde | 487-68-3 | C_10_H_12_O | 148.20 |
| Phenolic | 46 | Phenol | 108-95-2 | C_6_H_6_O | 94.11 |
|  | 47 | 5-isopropyl-2-methylphenol | 499-75-2 | C_10_H_14_O | 150.22 |
|  | 48 | 2,4-di-tert-butylphenol | 96-76-4 | C_14_H_22_O | 206.32 |
|  | 49 | P-cresol | 106-44-5 | C_7_H_8_O | 108.14 |
|  | 50 | 3-methyl-4-isopropylphenol | 3228-2-2 | C_10_H_14_O | 150.22 |
|  | 51 | Thymol | 89-83-8 | C_10_H_14_O | 150.22 |
| Organic Acids | 52 | Pentanoic acid, 5-hydroxy-, 2,4-bis(1,1-dimethylethyl) phenyl ester | 166273-38-7 | C_19_H_30_O_3_ | 306.44 |
|  | 53 | Dl-phenylsuccinic acid | 635-51-8 | C_10_H_10_O_4_ | 194.18 |
| Pyrazine | 54 | Tetramethylpyrazine | 1124-11-4 | C_8_H_12_N_2_ | 136.19 |
|  | 55 | 2,5-dimethyl-3-butylpyrazine | 40790-29-2 | C_10_H_16_N_2_ | 164.25 |
|  | 56 | 2,3,5-trimethylpyrazine | 14667-55-1 | C_7_H_10_N_2_ | 122.17 |
|  | 57 | 2,5-dimethyl-3-pentylpyrazine | 56617-69-7 | C_11_H_18_N_2_ | 178.27 |
|  | 58 | Pyrazine,3,5-dimethyl-2-(3-methylbutyl)- (9cl) | 111150-30-2 | C_11_H_18_N_2_ | 178.27 |
| Others | 59 | Naphthalene, decahydro-1-methyl-2-methylene | 90548-09-7 | C_12_H_20_ | 164.29 |
|  | 60 | Toluene | 108-88-3 | C_7_H_8_ | 92.14 |
|  | 61 | 1-(hexyloxy)-2-methylhexane | 74421-17-3 | C_13_H_28_O | 200.36 |
|  | 62 | 3,3-dimethylhexane | 563-16-6 | C_13_H_28_O | 200.36 |
|  | 63 | 4-octanone,3,5-dimethyl | 7335-17-3 | C_10_H_20_O | 156.27 |
|  | 64 | (r, r)- (+)-hydrobenzoin | 52340-78-0 | C_14_H_14_O_2_ | 214.26 |
|  | 65 | Ethyl ether | 60-29-7 | C_4_H_10_O | 74.12 |
|  | 66 | 1,2,4-trimethylbenzene | 95-63-6 | C_9_H_12_ | 120.19 |
|  | 67 | 5-phenyldodecane | 2719-63-3 | C_18_H_30_ | 246.43 |
|  | 68 | Styrene | 100-42-5 | C_8_H_8_ | 104.15 |
|  | 69 | 5-phenyldodecane | 2719-63-3 | C_18_H_30_ | 246.43 |
|  | 70 | Undecan-6-ylbenzene | 4537-14-8 | C_17_H_28_ | 232.40 |
|  | 71 | 1-methyl-2-(1-methylpentyl) cyclopropane | 62238-06-6 | C_10_H_20_ | 140.27 |
|  | 72 | 4-isopropylcyclohexanamine | 52430-81-6 | C_9_H_19_N | 141.25 |
|  | 73 | Chloromethyl isobutyl ether | 34180-11-5 | C_5_H_11_ClO | 122.59 |
|  | 74 | α-terpinene | 99-86-5 | C_10_H_16_ | 136.23 |
|  | 75 | 1-(hexyloxy)-3-methylhexane | 74421-18-4 | C_13_H_28_O | 200.36 |
|  | 76 | Pentane,2,2,3,4-tetramethyl | 1186-53-4 | C_9_H_20_ | 128.26 |

**Table S3** VIP value of volatile flavors in *Daqu* samples.

| Volatile flavors | Var ID (Primary) | M1.VIP [4] | 2.44693 * M1.VIP [4] cvSE |
| --- | --- | --- | --- |
| Benzene, 1-isocyano-3-methyl | 19 | 1.48923 | 1.64192 |
| 2-phenyl-5-methyl-2-hexenal | 43 | 1.3393 | 2.36683 |
| 2,4-dimethylbenzaldehyde | 45 | 1.31659 | 1.30554 |
| 2-methylundecan-4-one | 30 | 1.29742 | 1.08884 |
| Undecan-6-ylbenzene | 71 | 1.2811 | 2.04128 |
| Pyrrole-2-carboxaldehy | 42 | 1.27729 | 1.13576 |
| 2,5-dimethyl-3-pentylpyrazine | 58 | 1.2349 | 1.15179 |
| Mesitaldehyd | 46 | 1.22936 | 1.32405 |
| Ethyl pentadecanoate | 23 | 1.19428 | 1.2939 |
| 5-isopropyl-2-methylphenol | 48 | 1.14551 | 0.94508 |
| Dl-1-phenethylalcohol | 6 | 1.12228 | 0.958487 |
| 2,3,5-trimethylpyrazine | 57 | 1.10245 | 0.536435 |
| Tetramethylpyrazine | 55 | 1.09906 | 0.532028 |
| Toluene | 61 | 1.0759 | 0.661121 |
| 3-octanol | 1 | 1.06511 | 0.359609 |
| 2-cyclohexen-1-1,2,4,4-trimethyl-3-(3-methylbutyl) | 34 | 0.968773 | 0.879808 |
| Methyl 3-methylbenzoate | 21 | 0.965107 | 1.38399 |
| 1,2,4-trimethylbenzene | 67 | 0.962657 | 0.668853 |
| Nonan-1-ol | 3 | 0.937211 | 1.69414 |
| Benzaldehyde | 44 | 0.933888 | 0.824386 |
| Styrene | 69 | 0.924815 | 0.745419 |
| Pentane,2,2,3,4-tetramethyl | 77 | 0.903618 | 0.775413 |
| Pyrazine,3,5-dimethyl-2-(3-methylbut-yl)- (9Cl) | 59 | 0.854245 | 0.778371 |
| 2-ethylhexan-1-ol | 2 | 0.838528 | 0.84461 |
| 2,4-di-tert-butylphenol | 49 | 0.833708 | 1.15756 |
| Dl-phenylsuccinic acid | 54 | 0.803569 | 0.978517 |
| 2-propylheptan-1-ol | 4 | 0.802709 | 0.396056 |
| Acrolein | 39 | 0.776244 | 1.35396 |
| Propanoic acid, 2-methyl-, 2,2-dimethyl-1-(1-Methylethyl)-1,3-p-ropanediyl ester | 17 | 0.769957 | 0.514141 |
| Ethyl palmitate | 25 | 0.682898 | 0.726113 |
| 2,5-dimethyl-3-butylpyrazine | 56 | 0.655136 | 0.708533 |
| 2,5-hexanedione | 28 | 0.621489 | 0.601676 |
| 1-phenyl-5-methyl-1-hexanone | 38 | 0.494615 | 0.512988 |
| 2-butoxyethanol | 15 | 0.390262 | 0.460383 |
| Butyl acrylate | 16 | 0.195914 | 0.853223 |

**Table S4** Volatile flavor compounds identified in *Daqu* samples by GC-IMS.

| Compounds | CAS# | Formula | MW^a^ | RI^b^ | Rt^c^ [sec] | Dt^d^ [a.u.] | Identification approach |
| --- | --- | --- | --- | --- | --- | --- | --- |
| 4-Methyl-2-pentanol (internal standard) | C108112 | C_6_H_14_O | 102.2 | 752.5 | 243.436 | 1.564 | RI, DT |
| 1 | unidentified | * | 0.0 | 544.1 | 127.288 | 1.197 | RI, DT |
| 2 | unidentified | * | 0.0 | 565.0 | 135.806 | 1.212 | RI, DT |
| 3 | unidentified | * | 0.0 | 579.1 | 141.560 | 1.148 | RI, DT |
| 4 | unidentified | * | 0.0 | 581.9 | 142.711 | 1.170 | RI, DT |
| 5 | unidentified | * | 0.0 | 603.9 | 151.689 | 1.272 | RI, DT |
| 6 | unidentified | * | 0.0 | 602.7 | 151.229 | 1.293 | RI, DT |
| 7 | unidentified | * | 0.0 | 625.3 | 160.436 | 1.209 | RI, DT |
| 8 | unidentified | * | 0.0 | 628.6 | 161.818 | 1.242 | RI, DT |
| 9 | unidentified | * | 0.0 | 619.6 | 158.135 | 1.311 | RI, DT |
| 10 | unidentified | * | 0.0 | 625.8 | 160.667 | 1.361 | RI, DT |
| 11 | unidentified | * | 0.0 | 663.7 | 176.142 | 1.115 | RI, DT |
| 12 | unidentified | * | 0.0 | 694.5 | 191.552 | 1.100 | RI, DT |
| 13 | unidentified | * | 0.0 | 693.7 | 190.910 | 1.329 | RI, DT |
| 14 | unidentified | * | 0.0 | 693.4 | 190.589 | 1.367 | RI, DT |
| 15 | unidentified | * | 0.0 | 702.0 | 198.294 | 1.366 | RI, DT |
| 16 | unidentified | * | 0.0 | 710.3 | 205.677 | 1.266 | RI, DT |
| 17 | unidentified | * | 0.0 | 711.3 | 206.641 | 1.405 | RI, DT |
| 18 | unidentified | * | 0.0 | 709.6 | 205.035 | 1.456 | RI, DT |
| 19 | unidentified | * | 0.0 | 716.7 | 211.456 | 1.207 | RI, DT |
| 20 | unidentified | * | 0.0 | 723.9 | 217.877 | 1.169 | RI, DT |
| 21 | unidentified | * | 0.0 | 722.8 | 216.914 | 1.419 | RI, DT |
| 22 | unidentified | * | 0.0 | 737.9 | 230.397 | 1.174 | RI, DT |
| 23 | unidentified | * | 0.0 | 745.5 | 237.139 | 1.139 | RI, DT |
| 24 | unidentified | * | 0.0 | 745.8 | 237.460 | 1.168 | RI, DT |
| 25 | unidentified | * | 0.0 | 743.7 | 235.534 | 1.213 | RI, DT |
| 26 | unidentified | * | 0.0 | 744.8 | 236.497 | 1.368 | RI, DT |
| 27 | unidentified | * | 0.0 | 729.3 | 222.692 | 1.389 | RI, DT |
| 28 | unidentified | * | 0.0 | 751.2 | 242.276 | 1.516 | RI, DT |
| 29 | unidentified | * | 0.0 | 723.6 | 217.556 | 1.626 | RI, DT |
| 30 | unidentified | * | 0.0 | 754.1 | 244.844 | 1.325 | RI, DT |
| 31 | unidentified | * | 0.0 | 767.7 | 257.043 | 1.318 | RI, DT |
| 32 | unidentified | * | 0.0 | 774.6 | 263.143 | 1.276 | RI, DT |
| 33 | unidentified | * | 0.0 | 798.3 | 288.234 | 1.077 | RI, DT |
| 34 | unidentified | * | 0.0 | 797.4 | 287.126 | 1.425 | RI, DT |
| 35 | unidentified | * | 0.0 | 806.3 | 297.473 | 1.272 | RI, DT |
| 36 | unidentified | * | 0.0 | 805.0 | 295.995 | 1.450 | RI, DT |
| 37 | unidentified | * | 0.0 | 833.2 | 328.885 | 1.212 | RI, DT |
| 38 | unidentified | * | 0.0 | 832.6 | 328.146 | 1.284 | RI, DT |
| 39 | unidentified | * | 0.0 | 844.0 | 341.449 | 1.384 | RI, DT |
| 40 | unidentified | * | 0.0 | 871.5 | 373.600 | 1.235 | RI, DT |
| 41 | unidentified | * | 0.0 | 870.9 | 372.861 | 1.332 | RI, DT |
| 42 | unidentified | * | 0.0 | 871.5 | 373.600 | 1.403 | RI, DT |
| 43 | unidentified | * | 0.0 | 889.6 | 394.664 | 1.361 | RI, DT |
| 44 | unidentified | * | 0.0 | 894.6 | 402.055 | 1.387 | RI, DT |
| 45 | unidentified | * | 0.0 | 908.9 | 428.293 | 1.239 | RI, DT |
| 46 | unidentified | * | 0.0 | 910.3 | 430.731 | 1.453 | RI, DT |
| 47 | unidentified | * | 0.0 | 990.8 | 577.973 | 1.417 | RI, DT |
| 48 | unidentified | * | 0.0 | 1013.3 | 620.390 | 1.305 | RI, DT |
| 49 | unidentified | * | 0.0 | 1010.3 | 614.646 | 1.459 | RI, DT |
| 50 | unidentified | * | 0.0 | 990.8 | 577.881 | 1.850 | RI, DT |
| 51 | unidentified | * | 0.0 | 1135.6 | 853.653 | 1.342 | RI, DT |
| 1-propanol | C71238 | C_3_H_8_O | 60.1 | 563.0 | 135.013 | 1.250 | RI, DT |
| 2-butanone | C78933 | C_4_H_8_O | 72.1 | 592.3 | 146.974 | 1.248 | RI, DT |
| 2-methyl butanol dimer | C137326 | C_5_H_12_O | 88.1 | 746.9 | 238.423 | 1.481 | RI, DT |
| 2-methyl butanol monomer | C137326 | C_5_H_12_O | 88.1 | 744.4 | 236.176 | 1.241 | RI, DT |
| 2-methylbutanal | C96173 | C_5_H_10_O | 86.1 | 668.7 | 178.203 | 1.406 | RI, DT |
| 2-methylpropanal | C78842 | C_4_H_8_O | 72.1 | 563.9 | 135.345 | 1.277 | RI, DT |
| 2-methylpropanol | C78831 | C_4_H_10_O | 74.1 | 626.2 | 160.817 | 1.168 | RI, DT |
| 2-octanol | C123966 | C_8_H_18_O | 130.2 | 1005.4 | 605.367 | 1.441 | RI, DT |
| 3-methylbutanal dimer | C590863 | C_5_H_10_O | 86.1 | 655.7 | 172.861 | 1.414 | RI, DT |
| 3-methylbutanal monomer | C590863 | C_5_H_10_O | 86.1 | 651.8 | 171.278 | 1.168 | RI, DT |
| 3-octanone dimer | C106683 | C_8_H_16_O | 128.2 | 991.1 | 578.415 | 1.717 | RI, DT |
| 3-octanone monomer | C106683 | C_8_H_16_O | 128.2 | 991.1 | 578.415 | 1.305 | RI, DT |
| 5-Methyl-3-heptanone | C541855 | C_8_H_16_O | 128.2 | 954.2 | 510.952 | 1.284 | RI, DT |
| Acetoin | C513860 | C_4_H_8_O_2_ | 88.1 | 711.0 | 206.320 | 1.329 | RI, DT |
| Acetone | C67641 | C_3_H_6_O | 58.1 | 532.1 | 122.364 | 1.122 | RI, DT |
| Benzaldehyde | C100527 | C_7_H_6_O | 106.1 | 954.2 | 510.952 | 1.149 | RI, DT |
| Butyrolactone | C96480 | C_4_H_6_O_2_ | 86.1 | 912.7 | 435.145 | 1.085 | RI, DT |
| Ethanol | C64175 | C_2_H_6_O | 46.1 | 504.6 | 111.158 | 1.141 | RI, DT |
| Ethyl 2-hydroxypropanoate | C97643 | C_5_H_10_O_3_ | 118.1 | 805.6 | 296.734 | 1.543 | RI, DT |
| Ethyl 3-methylbutyrate dimer | C108645 | C_7_H_14_O_2_ | 130.2 | 844.0 | 341.449 | 1.658 | RI, DT |
| Ethyl 3-methylbutyrate monomer | C108645 | C_7_H_14_O_2_ | 130.2 | 844.6 | 342.188 | 1.257 | RI, DT |
| Ethyl acetate dimer | C141786 | C_4_H_8_O_2_ | 88.1 | 611.7 | 154.884 | 1.339 | RI, DT |
| Ethyl acetate monomer | C141786 | C_4_H_8_O_2_ | 88.1 | 611.1 | 154.664 | 1.095 | RI, DT |
| Ethyl acrylate | C140885 | C_5_H_8_O_2_ | 100.1 | 694.5 | 191.552 | 1.413 | RI, DT |
| Ethyl hexanoate | C123660 | C_8_H_16_O_2_ | 144.2 | 1010.0 | 614.052 | 1.338 | RI, DT |
| Furfurol dimer | C98011 | C_5_H_4_O_2_ | 96.1 | 822.1 | 315.950 | 1.334 | RI, DT |
| Furfurol monomer | C98011 | C_5_H_4_O_2_ | 96.1 | 822.4 | 316.320 | 1.082 | RI, DT |
| Geranyl acetate | C105873 | C_12_H_20_O_2_ | 196.3 | 1342.7 | 1248.920 | 1.221 | RI, DT |
| Hexanal dimer | C66251 | C_6_H_12_O | 100.2 | 790.2 | 278.707 | 1.565 | RI, DT |
| Hexanal monomer | C66251 | C_6_H_12_O | 100.2 | 790.2 | 278.707 | 1.252 | RI, DT |
| Hexanoic acid | C142621 | C_6_H_12_O_2_ | 116.2 | 980.0 | 558.090 | 1.302 | RI, DT |
| Isopentyl alcohol dimer | C123513 | C_5_H_12_O | 88.1 | 734.7 | 227.465 | 1.490 | RI, DT |
| Isopentyl alcohol monomer | C123513 | C_5_H_12_O | 88.1 | 730.6 | 223.870 | 1.237 | RI, DT |
| N-Nonanal | C124196 | C_9_H_18_O | 142.2 | 1137.7 | 857.693 | 1.472 | RI, DT |
| N-Nonanal | C124196 | C_9_H_18_O | 142.2 | 1103.3 | 792.076 | 1.470 | RI, DT |
| Phenylacetaldehyde dimer | C122781 | C_8_H_8_O | 120.2 | 1044.4 | 679.677 | 1.540 | RI, DT |
| Phenylacetaldehyde monomer | C122781 | C_8_H_8_O | 120.2 | 1043.3 | 677.599 | 1.253 | RI, DT |

^a^ Represent molecular mass.

^b^ Represents the retention index calculated using 4-Methyl-2-pentanol as external standard.

^c^ Represents the retention time in the capillary GC column.

^d^ Represents the drift time in the drift tube.

**Table S5** Polar metabolites contents (μmol/g) in 4 different *Daqu* samples (F-I-D; F-O-D; M-I-D; M-O-D).

| Presumptive metabolites and their contents (μmol/g) | | | | | |
| --- | --- | --- | --- | --- | --- |
| **Metabolites** | | F-I-D | F-O-D | M-I-D | M-O-D |
| Esters | 2-phosphoglycerate | nd | nd | nd | 2.6946±4.6672 |
|  | 2-hydroxyisobutyrate | nd | 0.0586±0.0727 | 0.0570±0.0527 | 0.0220±0.0381 |
|  | 3,4-dihydroxymandelate | nd | 0.0016±0.0028 | nd | 0.0333±0.0577 |
|  | 3-hydroxyphenylacetate | 0.0019±0.0020 | nd | nd | nd |
|  | 2-ethyl acrylate | nd | nd | 0.0030±0.0051 | 0.0946±0.0822 |
|  | Ethylmalonate | nd | nd | 0.0010±0.0017 | 0.0070±0.0121 |
|  | 1,3-dimethylurate | nd | 0.5873±0.2001 | 0.2740±0.3276 | 0.5310±0.2589 |
|  | 2-aminobutyrate | 0.0107±0.0110 | 0.1300±0.1125 | nd | nd |
|  | 2-Hydroxy-3-methylvalerate | nd | nd | 0.1663±0.2033 | nd |
|  | 2-phenylpropionate | nd | 5.9820±5.0134 | nd | nd |
|  | 2-oxoglutarate | nd | 0.1370±0.0985 | 0.0656±0.1137 | 0.0596±0.1033 |
|  | 3-hydroxyisovalerate | nd | 0.0016±0.0028 | nd | nd |
|  | 4-aminobutyrate | nd | 0.1180±0.2043 | nd | nd |
|  | Malonate | 0.0357±0.0365 | nd | nd | nd |
|  | Acetoacetate | 0.0382±0.0390 | 0.2380±0.4122 | nd | nd |
|  | Isobutyrate | nd | 0.0636±0.1102 | 0.4096±0.2346 | nd |
|  | Glycerate | 0.7871±0.1629 | 4.0293±0.7279 | 2.5250±0.8125 | 0.6290±1.0894 |
| Amino Acids & Derivatives | 3,5-dibromotyrosine | nd | 0.0536±0.0929 | 0.1970±0.3412 | nd |
|  | 4-carboxyglutamate | nd | 0.1973±0.3417 | nd | nd |
|  | 5-hydroxytryptophan | nd | 0.0523±0.0906 | nd | nd |
|  | N,n-dimethylglycine | nd | 0.0193±0.0334 | nd | nd |
|  | N-phenylacetylglycine | 0.6803±0.0937 | 0.4963±0.5926 | 1.1413±0.1344 | 0.7213±0.4955 |
|  | N-acetylglutamate | nd | nd | 0.0030±0.0051 | nd |
|  | Tyrosine | nd | nd | 0.0026±0.0046 | nd |
|  | Phenylalanine | nd | 0.0350±0.0606 | 0.0183±0.0317 | nd |
|  | Pyruvate | nd | 0.1853±0.1735 | nd | nd |
|  | Methionine | nd | nd | 0.0476±0.0825 | nd |
|  | Trans-4-Hydroxy-L-proline | nd | 0.3940±0.6824 | 0.4190±0.4251 | 0.2056±0.3562 |
|  | Glycylproline | nd | 0.0623±0.1079 | 0.8176±1.4162 | 0.4443±0.7696 |
|  | Sarcosine | nd | 0.0913±0.0837 | 0.0510±0.0612 | 0.0813±0.1408 |
|  | Saccharopine | nd | nd | 0.0400±0.0692 | nd |
|  | Threonate | 2.2982±0.3073 | 4.3090±3.7653 | 3.4783±0.9351 | 1.0440±1.3947 |
|  | Alloisoleucine | nd | 1.3336±0.3626 | 0.2093±0.3625 | 0.5813±1.0068 |
|  | Ibuprofen | nd | nd | 0.0426±0.0739 | nd |
|  | Γ-glutamylphenylalanine | 0.2160±0.0115 | nd | nd | nd |
|  | Histidine | nd | nd | 0.0103±0.0178 | nd |
| Alkalamides | N-acetylglutamine | nd | nd | nd | 0.6386±0.0654 |
|  | N-nitrosodimethylamine | 0.0246±0.0306 | 0.0786±0.1362 | 0.0476±0.0825 | nd |
|  | O-phosphocholine | nd | nd | 0.0516±0.0894 | nd |
|  | Carnitine | 0.1165±0.0422 | 0.0650±0.1125 | nd | nd |
|  | Trimethylamine N-oxide | 1.0893±0.5384 | 0.9733±0.651 | 3.0750±1.2457 | 5.2370±0.0706 |
|  | Ethanolamine | 0.0556±0.0964 | nd | 0.295±0.5109 | nd |
| Alcohols | Ethanol | 21.1194±2.251 | 18.7536±5.7176 | 29.7123±1.704 | 15.9543±1.8218 |
|  | Glycerol | 2.8439±0.5387 | 3.0120±1.3953 | 2.1640±2.2120 | 3.1786±2.9187 |
|  | Ethylene glycol | nd | 0.2133±0.2981 | 0.3023±0.3030 | 0.7536±0.0352 |
|  | Pyridoxine | 0.0754±0.0630 | 0.0583±0.1010 | nd | nd |
| Sugars & Derivatives | Udp-n-acetylglucosamine | nd | 0.0506±0.0877 | nd | 0.0186±0.0323 |
|  | UDP-galactose | nd | 0.0043±0.0075 | nd | 0.0616±0.1068 |
|  | Galactonate | nd | 0.0806±0.1397 | nd | 0.9236±1.2235 |
|  | Galactose | 0.7798±0.4219 | 0.5466±0.9468 | 0.1100±0.1905 | nd |
|  | Galactitol | nd | nd | 0.4883±0.4545 | nd |
|  | Mannose | 0.7327±0.7484 | 4.2970±2.1384 | 1.2446±0.3396 | 1.9593±0.4089 |
|  | Xylose | 0.3081±0.3146 | nd | 0.7050±1.2210 | nd |
|  | Glucose-1-phosphate | 0.0661±0.0675 | 0.3106±0.5380 | 0.2090±0.3619 | 1.9070±2.3306 |
|  | Glucitol | 1.0908±0.2377 | 1.6653±0.4615 | 1.9786±0.3628 | 1.7206±0.4038 |
|  | Glucuronate | nd | 1.1370±1.9693 | nd | nd |
|  | Lactulose | 0.3976±0.6887 | 0.7920±1.3717 | 0.0216±0.0375 | nd |
|  | Cellobiose | 0.3076±0.3141 | 0.4316±0.6654 | 0.1200±0.2078 | 0.9810±0.8621 |
|  | Glucarate | nd | nd | 0.7030±1.0704 | 0.4886±0.6133 |
|  | 3-phenyllactate | nd | nd | 0.0366±0.0635 | nd |
|  | Propionate | nd | nd | 0.1256±0.1683 | nd |
|  | Fumarate | nd | 0.0066±0.0115 | 0.4013±0.3476 | nd |
|  | Xanthurenate | nd | nd | 0.0116±0.0202 | nd |
|  | Creatine | nd | 0.0753±0.1012 | 0.0170±0.0294 | 0.0513±0.0406 |
|  | Adipate | nd | 0.0506±0.0877 | 0.0936±0.1185 | nd |
|  | Formate | 0.1929±0.1971 | 4.3196±0.6825 | 0.8923±0.7873 | 0.5510±0.1893 |
|  | Creatine phosphate | nd | 0.0160±0.0277 | nd | nd |
|  | S-sulfocysteine | 0.4526±0.1171 | 0.4883±0.5544 | 0.9313±0.8070 | 0.5366±0.9295 |
|  | Taurine | nd | 2.7793±0.6734 | 3.5373±0.3557 | 1.0660±1.8463 |
|  | Lactate | nd | 6.1826±0.8992 | 0.3710±0.5788 | 2.0513±1.1958 |
|  | Tropate | nd | nd | nd | nd |
|  | Caprylate | nd | nd | 0.0490±0.0848 | nd |
|  | Valerate | nd | 0.1070±0.1853 | nd | 0.0493±0.0854 |
|  | Glycolate | 0.0989±0.1010 | 1.1456±0.0822 | 0.9040±0.8246 | 1.6920±0.6679 |
|  | Protocatechuate | nd | 0.1156±0.2003 | 0.2113±0.2194 | 0.4233±0.0301 |
|  | Isovalerate | 0.0293±0.0200 | 0.3600±0.1270 | 0.8220±0.1637 | 0.6166±0.1375 |

^a^ Values represent means ± SD (n = 3).

^b^ nd, Not detected.

**Table S6** The value of Pearson correlation coefficients (Pearson correlation > |0.7|).

| Fig. 6 (a) | | | Fig. 6 (b) | | |
| --- | --- | --- | --- | --- | --- |
| microbiota | metabolites | Pearson correlation | microbiota | flavor compounds | Pearson correlation |
| A2 | D18 | 0.99286455 | A2 | M6 | 0.99521289 |
| B3 | E1 | 0.99185282 | A3 | M1 | 0.98753656 |
| A3 | H15 | 0.94875895 | A3 | I2 | 0.98734982 |
| A3 | H19 | 0.9398442 | A1 | N6 | 0.98458727 |
| A8 | D9 | 0.93876975 | A3 | L2 | 0.97757212 |
| A6 | H15 | 0.91793313 | A3 | K1 | 0.97662847 |
| B8 | H25 | 0.87923245 | A4 | K2 | 0.96473292 |
| A8 | G10 | 0.86776919 | A1 | N4 | 0.95813489 |
| A8 | D3 | 0.86776919 | B8 | J3 | 0.93762119 |
| A8 | H6 | 0.86776919 | A1 | M4 | 0.93658424 |
| A8 | H16 | 0.867769194 | B8 | I4 | 0.92900366 |
| B5 | H11 | 0.86704532 | B8 | I5 | 0.92791093 |
| A1 | H26 | 0.86332878 | A6 | M1 | 0.92616781 |
| A4 | C7 | 0.85334964 | B1 | J2 | 0.91860751 |
| B3 | E5 | 0.84694367 | B1 | M9 | 0.91308902 |
| A1 | E5 | 0.82776578 | A6 | L2 | 0.90933446 |
| A6 | H19 | 0.82280229 | A2 | M8 | 0.90706803 |
| B2 | H11 | 0.82104244 | A6 | K1 | 0.90667734 |
| A8 | H19 | 0.82066162 | A6 | I2 | 0.89233922 |
| A4 | H15 | 0.81333127 | B1 | M8 | 0.8867118 |
| A3 | G6 | 0.80155453 | A8 | I2 | 0.88590781 |
| A3 | H4 | 0.79154679 | B8 | I3 | 0.88436099 |
| A8 | G11 | 0.79102953 | B2 | I1 | 0.87700785 |
| B8 | H11 | 0.79017448 | A2 | M9 | 0.87520699 |
| B8 | G5 | 0.78614745 | A2 | M7 | 0.85805098 |
| B5 | D10 | 0.78491993 | A4 | L2 | 0.85514356 |
| B5 | H7 | 0.78491993 | B2 | I4 | 0.8479103 |
| B5 | H12 | 0.78491993 | B5 | I1 | 0.84612186 |
| B3 | F3 | 0.77669527 | B8 | I1 | 0.84273294 |
| A8 | G1 | 0.77534918 | A3 | N1 | 0.83712054 |
| B3 | C5 | 0.77084505 | A2 | J5 | 0.83178807 |
| B2 | D10 | 0.76806581 | B2 | I5 | 0.83140918 |
| B2 | H7 | 0.76806581 | A8 | M1 | 0.83058363 |
| B2 | H12 | 0.76806581 | B2 | J3 | 0.825375 |
| A3 | H2 | 0.76766439 | A8 | K1 | 0.82490605 |
| A8 | H4 | 0.76456494 | B3 | M3 | 0.82396046 |
| B8 | F1 | 0.76075062 | B2 | N6 | 0.82343077 |
| A4 | H18 | 0.75887634 | B2 | N4 | 0.82321524 |
| B2 | H25 | 0.75848041 | B5 | I4 | 0.82271366 |
| A8 | H2 | 0.75166407 | A1 | I1 | 0.80867802 |
| A3 | C7 | 0.74482312 | B5 | N6 | 0.808236 |
| A3 | D9 | 0.74203490 | A5 | N1 | 0.80464724 |
| A6 | G6 | 0.73717645 | B5 | N4 | 0.79877325 |
| A8 | H15 | 0.73376016 | B5 | I5 | 0.79731721 |
| B5 | G13 | 0.73240601 | A8 | L2 | 0.79473917 |
| B8 | D10 | 0.72545308 | A3 | K2 | 0.79411155 |
| B8 | H7 | 0.72545308 | B5 | J3 | 0.79311179 |
| B8 | H12 | 0.72545308 | B2 | I3 | 0.78272844 |
| A2 | C4 | 0.71788704 | A6 | N1 | 0.7791909 |
| B2 | G13 | 0.71256234 | A4 | K1 | 0.77442672 |
| A8 | D8 | 0.70947434 | A8 | N1 | 0.76008388 |
| B2 | H26 | 0.70930039 | A5 | J2 | 0.74507004 |
| A2 | H9 | 0.70794052 | B5 | I3 | 0.74336407 |
| A6 | D2 | 0.70211431 | A4 | M1 | 0.72461664 |
| A6 | D4 | 0.70211431 | B1 | N1 | 0.72185781 |
| A6 | H8 | 0.70211431 | A6 | K2 | 0.71348815 |
| A2 | H23 | -0.70088207 | A4 | I2 | 0.71343001 |
| A7 | D5 | -0.71560396 | B3 | M2 | 0.71017956 |
| B1 | H26 | -0.74336571 | A1 | M2 | 0.70910284 |
| A4 | D18 | -0.76482798 | B1 | I1 | -0.7149904 |
| B1 | C8 | -0.77887221 | B5 | J2 | -0.7168074 |
| A2 | H26 | -0.81521970 | B1 | M2 | -0.7281743 |
| B1 | E5 | -0.84069242 | B2 | J2 | -0.7378064 |
|  |  |  | A2 | M5 | -0.7584963 |
|  |  |  | A4 | M6 | -0.7631308 |
|  |  |  | A1 | N1 | -0.768882 |
|  |  |  | A5 | N2 | -0.7809431 |
|  |  |  | A5 | N6 | -0.8003453 |
|  |  |  | A4 | J5 | -0.8179683 |
|  |  |  | A4 | J4 | -0.8288584 |
|  |  |  | A5 | N4 | -0.8308459 |
|  |  |  | A8 | N2 | -0.8317518 |
|  |  |  | A2 | M4 | -0.8934838 |
|  |  |  | B1 | M4 | -0.8999637 |
|  |  |  | A6 | N2 | -0.8999876 |
|  |  |  | B1 | N4 | -0.9205533 |
|  |  |  | A1 | M8 | -0.9259326 |
|  |  |  | A3 | N2 | -0.9403062 |
|  |  |  | A1 | M9 | -0.9423351 |
|  |  |  | B1 | N6 | -0.9433363 |
|  |  |  | A4 | M7 | -0.9458915 |
|  |  |  | A1 | J2 | -0.9516565 |

**Table S7** Summary of sequencing results of wheat and the top 20 genera detected in wheat

| Type | Genus | Wheat1 | Wheat2 | Wheat3 | Wheat4 |
| --- | --- | --- | --- | --- | --- |
| Top genus （bacteria, %） | *Staphylococcus* | 0.04923456 | 0.0745029 | 0.05792715 | 0.04286468 |
|  | *Saccharopolyspora* | 0.00042231 | 0.00059828 | 0.00035193 | 0.00278022 |
|  | *Unidentified* | 0.04592645 | 0.07633292 | 0.05366884 | 0.04261834 |
|  | *Pantoea* | 0.36389231 | 0.21889847 | 0.31884568 | 0.40900933 |
|  | *Kroppenstedtia* | 0.01238782 | 0.01354918 | 0.00805912 | 0.01963752 |
|  | *Bacillus* | 0.10614112 | 0.08192856 | 0.08872075 | 0.03016013 |
|  | *Enterobacter* | 0.07862045 | 0.09882105 | 0.09804681 | 0.1225761 |
|  | *Acinetobacter* | 0.06112969 | 0.06880169 | 0.05581559 | 0.07492522 |
|  | *Sphingobacterium* | 0.05215555 | 0.05106458 | 0.07063171 | 0.0658455 |
|  | *Chryseobacterium* | 0.05715291 | 0.09100827 | 0.06264297 | 0.02822453 |
|  | *Lentibacillus* | 7.04E-05 | 7.04E-05 | 0 | 7.04E-05 |
|  | *Enterococcus* | 0.01960232 | 0.03100475 | 0.02512757 | 0.02526834 |
|  | *Lactobacillus* | 0.00066866 | 0.00218195 | 0.00147809 | 0.00031673 |
|  | *Saccharomonospora* | 0 | 7.04E-05 | 0 | 3.52E-05 |
|  | *Sphingomonas* | 0.01485131 | 0.01886328 | 0.01647017 | 0.01228225 |
|  | *Delftia* | 0.01502727 | 0.01309168 | 0.01400669 | 0.01594228 |
|  | *Brevibacterium* | 0.00017596 | 0.00024635 | 0.00035193 | 0.00052789 |
|  | *Stenotrophomonas* | 0.00777758 | 0.01013549 | 0.0110505 | 0.00805912 |
|  | *Pseudomonas* | 0.0101003 | 0.00450466 | 0.00531409 | 0.01520324 |
|  | *Bromus_tectorum* | 0.00376562 | 0.01210628 | 0.00629949 | 0.00137251 |
|  | *Other* | 0.10089741 | 0.1322189 | 0.10519092 | 0.08228049 |
| Top genus (fungi, %） | *Fusarium* | 0.09276115 | 0.10406231 | 0.06169823 | 0.1200672 |
|  | *Thermoascus* | 0.06841784 | 0.06771533 | 0.00222969 | 0.00409285 |
|  | *Byssochlamys* | 0.00073305 | 0.00448992 | 0.0021686 | 0.00015272 |
|  | *Unidentified* | 0.22947465 | 0.2872022 | 0.2840562 | 0.27229688 |
|  | *Aspergillus* | 0.11572999 | 0.15510079 | 0.14108125 | 0.20983506 |
|  | *Sporobolomyces* | 0.13066585 | 0.10852169 | 0.14871717 | 0.10665852 |
|  | *Trichosporon* | 0.14447159 | 0.05739157 | 0.10620037 | 0.03109346 |
|  | *Microascus* | 0.00226023 | 0.00149664 | 0.0007025 | 0 |
|  | *Thermomyces* | 0.00054979 | 0.00174099 | 0.00253513 | 0 |
|  | *Wallemia* | 0.01963958 | 0.02919976 | 0.02599267 | 0.05546732 |
|  | *Mortierella* | 0.00161881 | 0.0111179 | 0.00109957 | 0.0048259 |
|  | *Saitozyma* | 0.00769701 | 0.00626145 | 0.00302382 | 0.00061087 |
|  | *Holtermanniella* | 0.03759927 | 0.01093464 | 0.01551619 | 0.01759316 |
|  | *Penicillium* | 0.0113317 | 0.01686011 | 0.00784973 | 0.0084606 |
|  | *Cutaneotrichosporon* | 0.01985339 | 0.00717776 | 0.01789859 | 0.02663409 |
|  | *Vishniacozyma* | 0.00613928 | 0.01667685 | 0.01985339 | 0.02645082 |
|  | *Apiotrichum* | 0.02135003 | 0.01408064 | 0.03078803 | 0 |
|  | *Filobasidium* | 0.02089188 | 0.00555895 | 0.00763592 | 0.02409896 |
|  | *Cryptococcus* | 0.00971289 | 0.00751374 | 0.01136225 | 0.02446549 |
|  | *Candida* | 0.01194258 | 0.0099267 | 0.02131949 | 0.00937691 |
|  | *Other* | 0.04715944 | 0.07697007 | 0.08827123 | 0.05781918 |
